# An enhanced single phase quasi Z-source switched capacitor seven level inverter with modified SPWM for PV applications

**DOI:** 10.1038/s41598-025-94209-5

**Published:** 2025-03-22

**Authors:** Semko Hosseini, Seyed Hossein Hosseini, Mehran Sabahi, Ebrahim Babaei

**Affiliations:** 1 Faculty of Electrical and Computer Engineering, University of Tabriz, Tabriz, 51666-16471 Iran; 2 Engineering Faculty, Near East University, 99138 Nicosia, Turkey

**Keywords:** PV inverter, Symmetric, Leakage current, Quasi-Z-source (qZS) network, Shoot-through (ST) state, Switched capacitor multi-level inverter (SC-MLI), Electrical and electronic engineering, Renewable energy

## Abstract

The combination of impedance source networks with switched capacitor multilevel inverters (SC-MLIs) can address the inrush current problem, enhance voltage-gain, and improve immunity which is came from Z-source topology. This paper proposes an improved symmetric single-phase transformerless quasi-Z-Source based on switched capacitor 7-Level inverter (qZ-SC7LI) with a modified modulation technique. The circuit configuration of the qZ-SC7LI is meticulously designed to completely eradicate the issue of leakage current. To address the limited number of shoot-through (ST) states at the zero-voltage level in the proposed topology, a modified modulation technique has been developed. This technique creates ST states at various other voltage levels. Implicitly, this technique reduces the variation of the inverter output voltage (Δv). Furthermore, the qZ-SC7LI can handle reactive power, and continuously derive current from input DC source. Owing to developing stair case output voltage and needing low value for ST duty-ratio (d) the total harmonic distortion (THD) of injected current to the grid is improved and filter size is reduced. The proposed topology is verified by simulation in PSIM software. For experimentally verification of the proposed topology properties, related studies on a 1.2kVA-Lab-scale prototype are carried out.

## Introduction

The remarkable features of switched capacitor multi-level inverters (SC-MLIs), such as their voltage boost capability, self-balancing of capacitor voltages, and straightforward structure with minimal components, render them well-suited for circuit configuration powered by low voltage renewable energy sources like Photovoltaic (PV) arrays and fuel cells^1–3^. Hence a variety of SC-MLI topologies have emerged recently^4–6^. These types of inverters have also gained popularity in various power system research fields, such as Microgrids, photovoltaic grid tied applications^7,8^. On the other hand, their voltage-gain remains constant, and is not adjustable. The voltage-gain may also be affected by factors such as the efficiency of the switching process and the presence of parasitic components^9,10^. As a solution to this issue, in reference^11^, the use of an isolated dc-dc converter was recommended, however this approach results in increased circuit complexity and additional losses^12^.

Another prevalent challenge in SC-MLIs is the inrush current that occurs during capacitor charging^13,14^; although the addition of a limiting inductor to the SC-MLIs has been suggested as a solution in references^15,16^, the resulting resonant operation leads to the complexity of the inverter design and control, while the overall voltage-gain is not flexible.

To address mentioned constraints, combining a suitable impedance source (ZS) or quasi-impedance source (qZS) network as a voltage regulator with an appropriate topology of SC-MLI can be a trust worthy solution. The combination of these networks with inverters can bring the ability to increase voltage-gain, prevent electro-magnetic interference (EMI) and more immunity caused by the possibility of short circuit in some switch-legs, which is called ST-immunity^17–19^.

The aforementioned combination, when used in transformerless (non-isolated) grid-tied applications for PV systems, must adhere to the limits specified by valid standards for reducing leakage current. In this context, the criterion for reducing the leakage current in most of the cases in the literature is the standard VDE 0126-1-1 which specifies that the leakage current should be kept below a certain limit (300 mA peak to peak)^20^.

To overcome inrush current issue, a qZS-based symmetric three-phase and asymmetric single-phase inverter topologies are suggested in reference^21^. While the three-phase topology in this reference is ideal for three-phase PV applications due to its symmetrical structure that attenuates leakage current, the single-phase topology cannot mitigate leakage current because of its asymmetric structure. So the single-phase topology in this reference cannot be inverter of choice for grid-tied PV application. Furthermore, the asymmetric structure results in a DC-offset in inverter output voltage^22,23^. Moreover, each individual phase unit of the symmetric three-phase topology presented in reference^24^ cannot function as an independent single-phase PV inverter due to the constraints of its modulation technique and setup. These constraints arise from the modulation^’^s inability to create ST states for each single-phase unit separately.

**Fig. 1:**
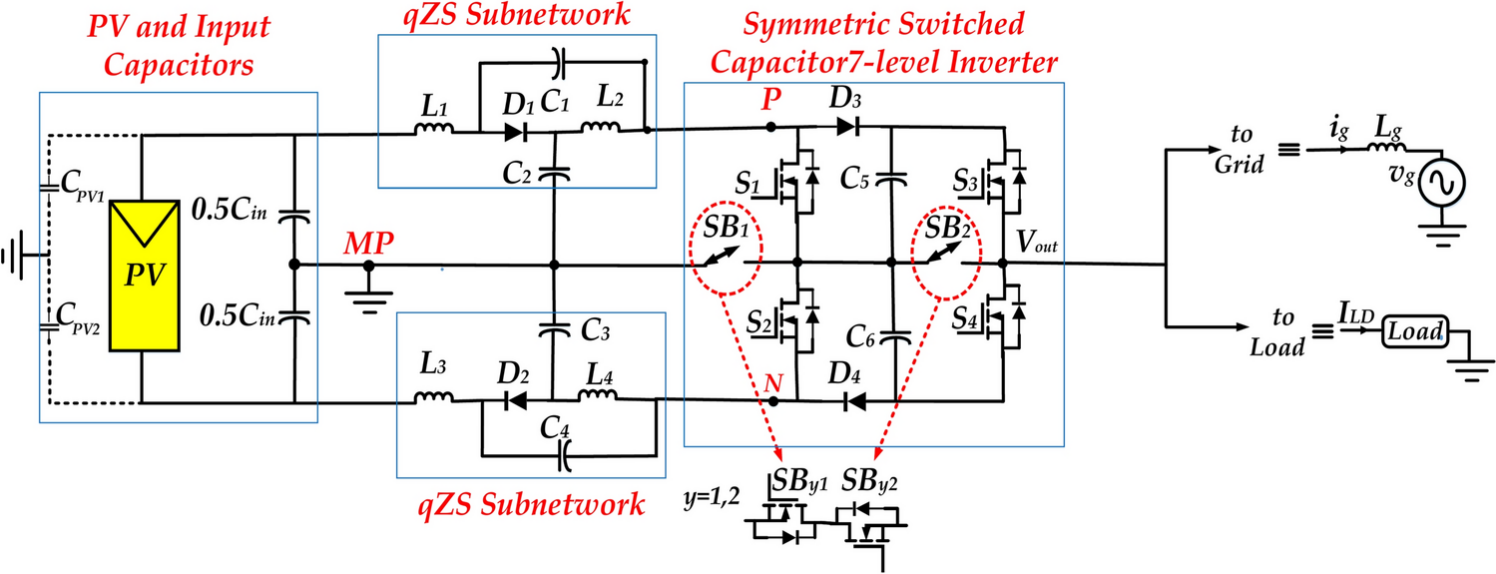
A schematic of proposed topology (qZ-SC7LI).

In light of above, in this paper, a topology for single-phase qZS based Switched Capacitor 7-Level Inverter (qZ-SC7LI), as shown in Fig. 1, is proposed. This topology is a combination of double qZS sub-network with a symmetric 7-level SC-MLI. As depicted in Fig. 1, each qZS sub-network includes one diode, two capacitors, and two inductors. Additionally, the symmetric 7-level SC-MLI includes four unidirectional power switches *(S*_*1*_*-S*_*4*_*)*, two bidirectional switches *(SB*_*1*_*, SB*_*2*_*)*, two diodes *(D*_*3*_*, D*_*4*_*)*, and two capacitors *(C*_*5*_*, C*_*6*_*)*.

Furthermore, ST state is created by simultaneously turning on three power switches: *S*_*1*_, *S*_*2*_, and *SB*_*1*_. The DC-side has a relatively high boost-factor by low value of ST duty- ratio (*d)*, beside an improve modulation strategy is proposed to prevent the adverse effect of ST states on the quality of the output voltage. Therefore this inverter can develop a high-quality (low THD) boosted 7 level AC output. In this proposed topology the leakage current, caused by the photovoltaic parasitic capacitors *(C*_*PV1,*_* C*_*PV2*_*),* is attenuated effectively by symmetrically clamping two input capacitors and two qZS sub-networks to the middle point (MP).

By employing the proposed modified switching strategy, the inverter provides a set of seven voltage levels at its output with the possibility of creating the ST state (by simultaneously turning on the switches *S*_*1*_, *S2,* and *SB1)*. Notably, the ST state is achievable at the zero and two other active voltage levels.

Briefly, the benefits of the proposed topology are as follow:

(I) Seven-level staircase AC voltage with lower THD and dv/dt.
(II) Attenuating parasitic leakage current in grid-tied PV application.
(III) Alleviating capacitor inrush current.
(IV) Achieving high voltage gain through two stage of ST and switched-capacitor concept.
(V) modified modulation technique to improve shoot through to make ST in different voltage levels
(VI) Modified modulation strategy which reduces voltage ripples across the capacitors.
(VII) Continuity of drawing current from the input source which eases MPPT (Maximum Power Point Tracking) process.

The rest of this paper is structured as follows: the proposed topology principles including mathematical model of qZS network, operation concept, the proposed modified SPWM technique, and passive components selection are described in the next section; Sect. "Voltage across parasitic capacitors & leakage current" deals with the leakage current issue. The explanation for power loss and efficiency calculations can be found in Sect. "Power loss & efficiency calculation". In Sect. "Simulation and discussion" the proposed topology is simulated in grid-tied PV application using P&O MPPT algorithm. The comparison with other state-of-the-art topologies is carried out in Sect. "Comparison with pervious topologies". In Sect. "Experimental results", the experimental results obtained from employing a lab-scale prototype are presented. These results are detailed in a stand-alone case study, which highlights the prototype’s efficacy. Furthermore, a comparative analysis between the proposed topology and other existing topologies is provided. Finally, Sect. "Conclusions" is allocated to overall work conclusion.

## Proposed topology principle

In this section, mathematic model of the DC-side (qZS network), the operation principle of the AC-side, the implementation of the modified SPWM technique and the passive components selection are described, respectively.

### Mathematical model of DC-side

**Fig. 2:**
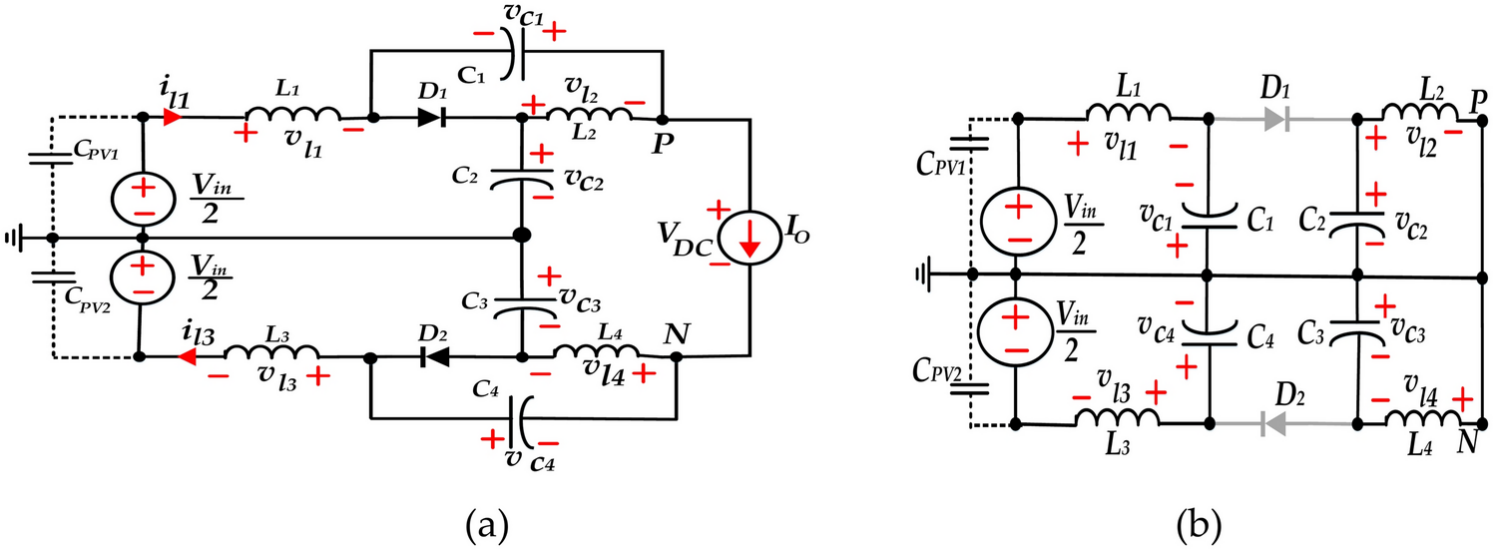
Model of qZS network, (**a**) In NST state, and (**b**) In ST state.

The equivalent circuits of NST and ST states of the qZS network utilized in the proposed topology are shown in Fig. 2. For simplicity in Fig. 2a, the PV array, input capacitors and the inverter are represented with two DC source by voltage value of *(V*_*in*_*/2)* and a current source *(I*_*o*_*)*, respectively.

Due to the symmetry of the qZS network, the following relations are obtained as follows:

1 $$\left\{ \begin{gathered} L_{1} = L_{3} ,L_{2} = L_{4} \hfill \\ C_{1} = C_{4} ,C_{2} = C_{3} \hfill \\ \end{gathered} \right. \Rightarrow \left\{ \begin{gathered} v_{l1} = v_{l3} ,v_{l2} = v_{l4} \hfill \\ V_{C1} = V_{C4} ,V_{C2} = V_{C3} \, \hfill \\ \end{gathered} \right.$$

By using a modified SPWM that will be discussed in the next section, the ST states are uniformly distributed during a fundamental period *(T)*. Therefore, in (2), voltage and current balance laws can be expressed for the corresponding passive components of the qZS network, over one *T*.

2 $$\int\limits_{T} {v_{lk} } dt = 0,\int\limits_{T} {i_{Ck} } dt = 0{ ; }k \in {\text{\{ 1,2,3,4\} ,T = T}}_{{{\text{ST}}}} {\text{ + T}}_{{{\text{NST}}}} {; }d = \frac{{{\text{T}}_{{{\text{ST}}}} }}{{\text{T}}}$$

where *v*_*l1*_, *v*_*l2*_, *v*_*l3*_, *v*_*l4*_, *i*_*C1*_, *i*_*C2*_, *i*_*C3*_, and *i*_*C4*_ represent the voltage across inductors *L*_*1*_*, L*_*2*_,* L*_*3*_,* L*_*4*_ and the current through capacitors *C*_*1*_,* C*_*2*_,* C*_*3*_, and *C*_*4*_ in (2), respectively. By applying the voltage balance law for the inductors of the qZS network and the current balance law for the capacitors of the qZS network, relations (3) to (10) are obtained.

3 $$\, \int\limits_{T} {v_{l1} } dt = 0 \, \Rightarrow \, (\frac{{V_{in} }}{2} - V_{C2} )(T - T_{ST} ) + (\frac{{V_{in} }}{2} + V_{C1} )(T_{ST} ) = 0 \Rightarrow (\frac{{V_{in} }}{2} - V_{C2} )(1 - d) + (\frac{{V_{in} }}{2} + V_{C1} )(d) = 0$$

4 $$\, \int\limits_{T} {v_{l2} } dt = 0 \, \Rightarrow \, ( - V_{C1} )(T - T_{ST} ) + (V_{C2} )(T_{ST} ) = 0 \Rightarrow ( - V_{C1} )(1 - d) + (V_{C2} )(d) = 0$$

5 $$\, \int\limits_{T} {v_{l3} } dt = 0 \, \Rightarrow \, (\frac{{V_{in} }}{2} - V_{C3} )(T - T_{ST} ) + (\frac{{V_{in} }}{2} + V_{C4} )(T_{ST} ) = 0 \Rightarrow \, (\frac{{V_{in} }}{2} - V_{C3} )(1 - d) + (\frac{{V_{in} }}{2} + V_{C4} )(d) = 0$$

6 $$\, \int\limits_{T} {v_{l4} } dt = 0 \, \Rightarrow \, ( - V_{C4} )(T - T_{ST} ) + (V_{C3} )(T_{ST} ) = 0 \Rightarrow \, ( - V_{C4} )(1 - d) + (V_{C3} )(d) = 0$$

7 $$\, \int\limits_{T} {i_{C1} } dt = 0 \, \Rightarrow \, (I_{L2} - I_{O} )(T - T_{ST} ) + ( - I_{L1} )(T_{ST} ) = 0 \Rightarrow \, (I_{L2} - I_{O} )(1 - d) + ( - I_{L1} )(d) = 0$$

8 $$\, \int\limits_{T} {i_{C2} } dt = 0 \, \Rightarrow \, (I_{L1} - I_{O} )(T - T_{ST} ) + ( - I_{L2} )(T_{ST} ) = 0 \Rightarrow \, (I_{L1} - I_{O} )(1 - d) + ( - I_{L2} )(d) = 0$$

9 $$\, \int\limits_{T} {i_{C3} } dt = 0 \, \Rightarrow \, (I_{L3} - I_{O} )(T - T_{ST} ) + ( - I_{L4} )(T_{ST} ) = 0 \Rightarrow \, (I_{L3} - I_{O} )(1 - d) + ( - I_{L4} )(d) = 0$$

10 $$\, \int\limits_{T} {i_{C4} } dt = 0 \, \Rightarrow \, (I_{L4} - I_{0} )(T - T_{ST} ) + ( - I_{L3} )(T_{ST} ) = 0 \Rightarrow \, (I_{L4} - I_{0} )(1 - d) + ( - I_{L3} )(d) = 0$$

where *V*_*C1*_*, V*_*C2*_,* V*_*C3*_, and *V*_*C4*_ denote the average voltages across capacitors *C*_*1,*_* C*_*2,*_* C*_*3*_ and *C*_*4*_ over one fundamental period *(T)* and *I*_*L1*_*, I*_*L2*_,* I*_*L3*_, and* I*_*L4*_ represent the average currents through inductors *L*_*1,*_* L*_*2,*_* L*_*3*_ and *L*_*4*_ over the same period. These values are provided in Eqs. (3–10), respectively.

Consequently, estimated values of the average voltage across the capacitors *C*_*1*_ to *C*_*4*_and the average current through the inductors* L*_*1*_ to *L*_*4*_, outlined below:

11 $$V_{C1} = V_{C4} = \frac{{dV_{in} }}{2 - 4d}, \, V_{C2} = V_{C3} = \frac{{(1 - d)V_{in} }}{2 - 4d}$$

12 $$I_{L1} = I_{L2} = I_{L3} = I_{L4} = \frac{{\left( {1 - d} \right)I_{o} }}{1 - 2d}$$

As illustrated in Fig. 2a, the average voltage between points ‘*P*’, and ’*N*’ over one fundamental period (*T)* can be calculated as:

13 $$V_{DC} = V_{C1} + V_{C2} + V_{C3} + V_{C4}$$

Therefore, the qZS boost-factor (*G*) can be obtained as follows:

14 $$V_{DC} = \frac{{V_{in} }}{1 - 2d} \Rightarrow G = \frac{{V_{DC} }}{{V_{in} }} = \frac{1}{1 - 2d}$$

The symmetric 7-level SC-MLI in the proposed topology inherently achieves a maximum voltage gain of 1.5, which will be discussed in the subsequent section. Consequently, the following relationship can be derived:

15 $$Vout = 1.5mV_{DC} \Rightarrow Vout = \frac{{1.5mV_{in} }}{1 - 2d},B = \frac{1.5}{{1 - 2d}}$$

where *V*_*out*_*, V*_*DC*_,* m*, *V*_*in*_*, d*, and *B* denote the output voltage of the proposed topology, average voltage between points ‘*P*’, and ’*N*’ over one fundamental period (*T*), modulation index, input voltage of the proposed topology, shoot-through duty ratio, and the overall boost-factor of the proposed topology in(15), respectively.

### Operational concept of AC-side

**Fig. 3:**
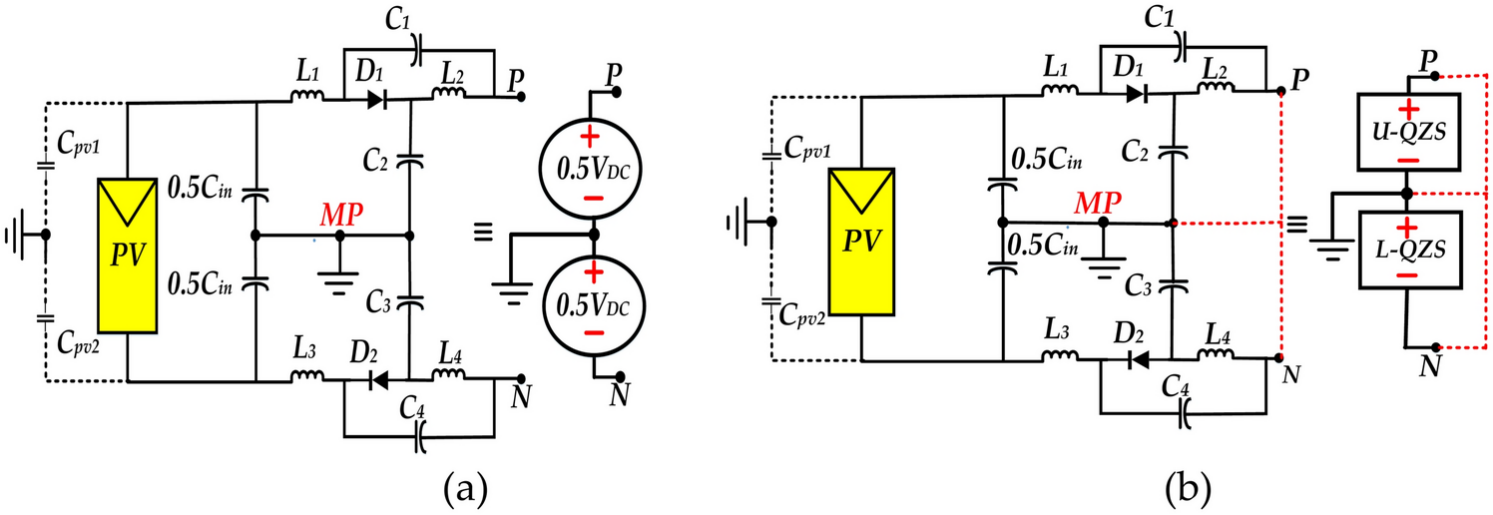
Representation of DC-side of the proposed topology, (**a**) In NST state, and (**b**) In ST state.

**Fig. 4:**
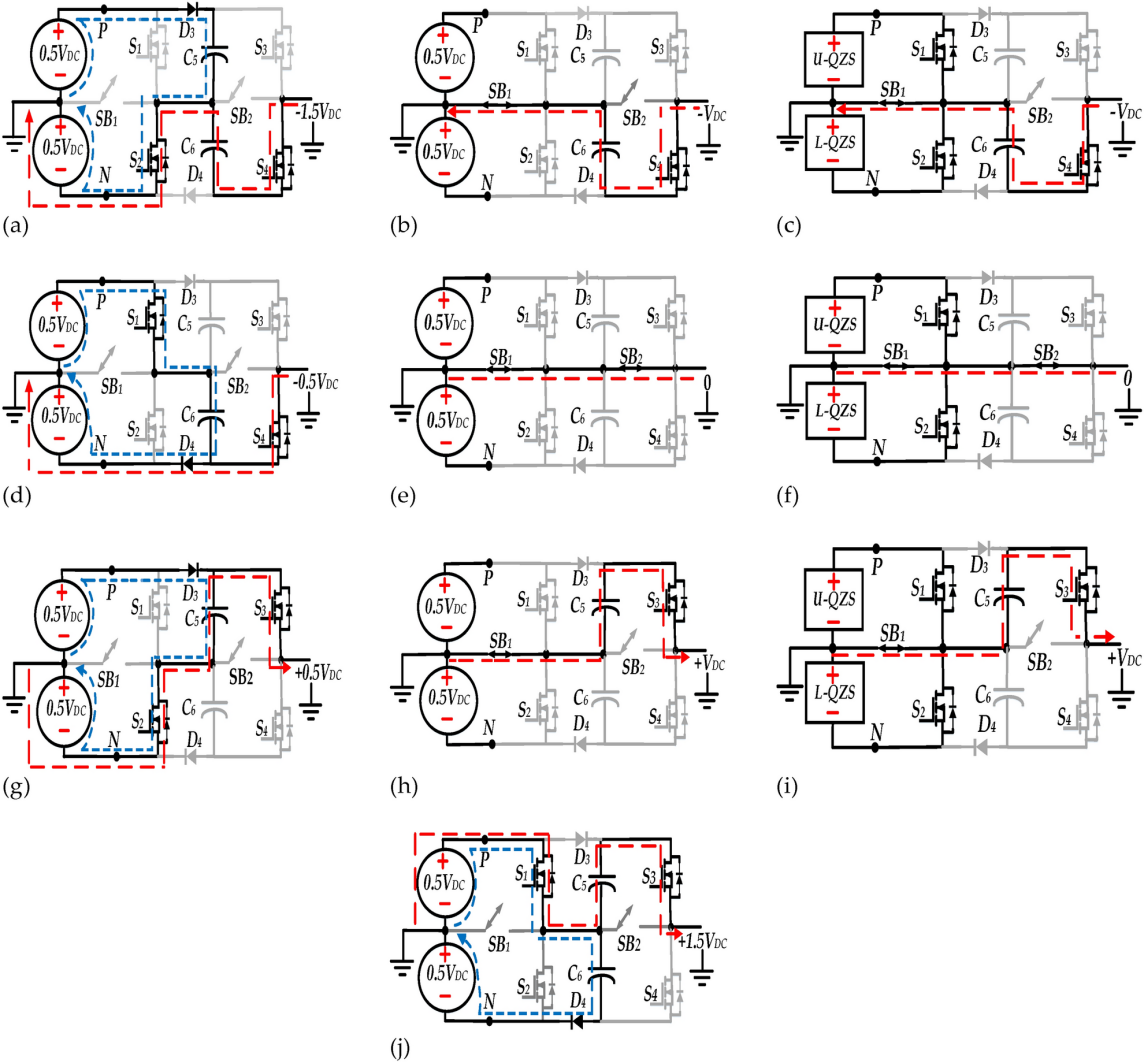
Switching modes of the qZS-7LI, (**a**) First Level, (**b**) Second Level at NST state, (**c**) Second Level at ST state, (**d**) Third Level, (**e**) Fourth Level at NST state, (**f**) Fourth Level at ST state, (**g**) Fifth Level, (**h**) Sixth Level at NST state, (**i**) Sixth Level at ST state, and (**j**) Seventh Level.

To facilitate the explanation of this section, the DC-side as shown in Fig. 3, is taken into consideration. In this figure, the DC-side of the topology is represented by two connected DC sources of *0.5V*_*DC*_, also by the boxes labeled *U-QZS* and *L-QZS* in None-ST (NST) and ST modes, respectively. The AC-side of the proposed topology is consisted of four active power switches *(S*_*1*_* to S*_*4*_*)* and two bidirectional switches *(SB*_*1*_*, SB*_*2*_*)*. In order to descript as depicted in Fig. 4a to j, switching modes result in seven voltage levels *(LV*_*1*_* to LV*_*7*_*)* at the inverter output. Additionally current path of the inverter output and charging current path of capacitors *C*_*5*_ and *C*_*6*_ are indicated by dashed red and blue lines, respectively.

Referring to Fig. 4a,d,g and j the capacitors *C*_*5*_ and *C*_*6*_ are charged to *V*_*DC*_, so it is possible to provide six voltage levels, by adding and subtracting output voltage of *U-QZS* or *L-QZS* and the capacitors *C*_*5*_ and *C*_*6*_ voltages, resulting in a staircase voltage wave in the inverter output. Moreover as depicted in Fig. 4e and f, for providing zero voltage level (*LV*_*4*_), at the NST state two bidirectional switches (*SB*_*1*_*, SB*_*2*_) are turned on simultaneously, however at ST state for developing this level, switches(*S*_*1*_,*S*_*2*_) and bidirectional switches (*SB*_*1*_*, SB*_*2*_) must be turned on.

Furthermore, Table 1 tabulates the occurrence of the ST state, the switching state of switches *S*_*1*_,* S*_*2*_, *S*_*3*_, *S*_*4*_, *SB*_*1*_, *SB*_*2*_, and diodes *D*_*3*_,* D*_*4*_ for each voltage level and the charging/discharging modes of the capacitors *C*_*5*_,* C*_*6*_.

**Table 1:** Operational states.

Level	ST	*S*_*1*_*,S*_*2*_*,S*_*3*_*,S*_*4*_*,SB*_*1*_*,SB*_*2*_	D_1_, D_2_	D_3_, D_4_	C_5_, C_6_	Vout
1	0	010100	11	10	C, D	*− 1.5V*_*DC*_
2	0	000110	11	00	N, D	*− V*_*DC*_
	1	110,110	00	00	N, D	*− V*_*DC*_
3	0	100,100	11	01	N, C	*− 0.5V*_*DC*_
4	0	000011	11	00	N, N	*0*
	1	110,011	00	00	N, N	*0*
5	0	011000	11	10	C, N	+ *0.5V*_*DC*_
6	0	001010	11	00	D, N	+ *V*_*DC*_
	1	111,010	00	00	D, N	+ *V*_*DC*_
7	0	101,000	11	01	D, C	+ *1.5V*_*DC*_

(i) The occurrence of the ST state and NST state in second column are indicated by ‘1’, and ‘0’ respectively. (ii) The “on” and “off” state of the switches and diodes are indicated by ‘1’, and ‘0’respectively. (iii) The charging, discharging and non-connecting modes of the capacitors (*C*_*5*_*&C*_*6*_) are indicated by "C', "D" and "N", respectively.

### Proposed modified SPWM modulation

**Fig. 5:**
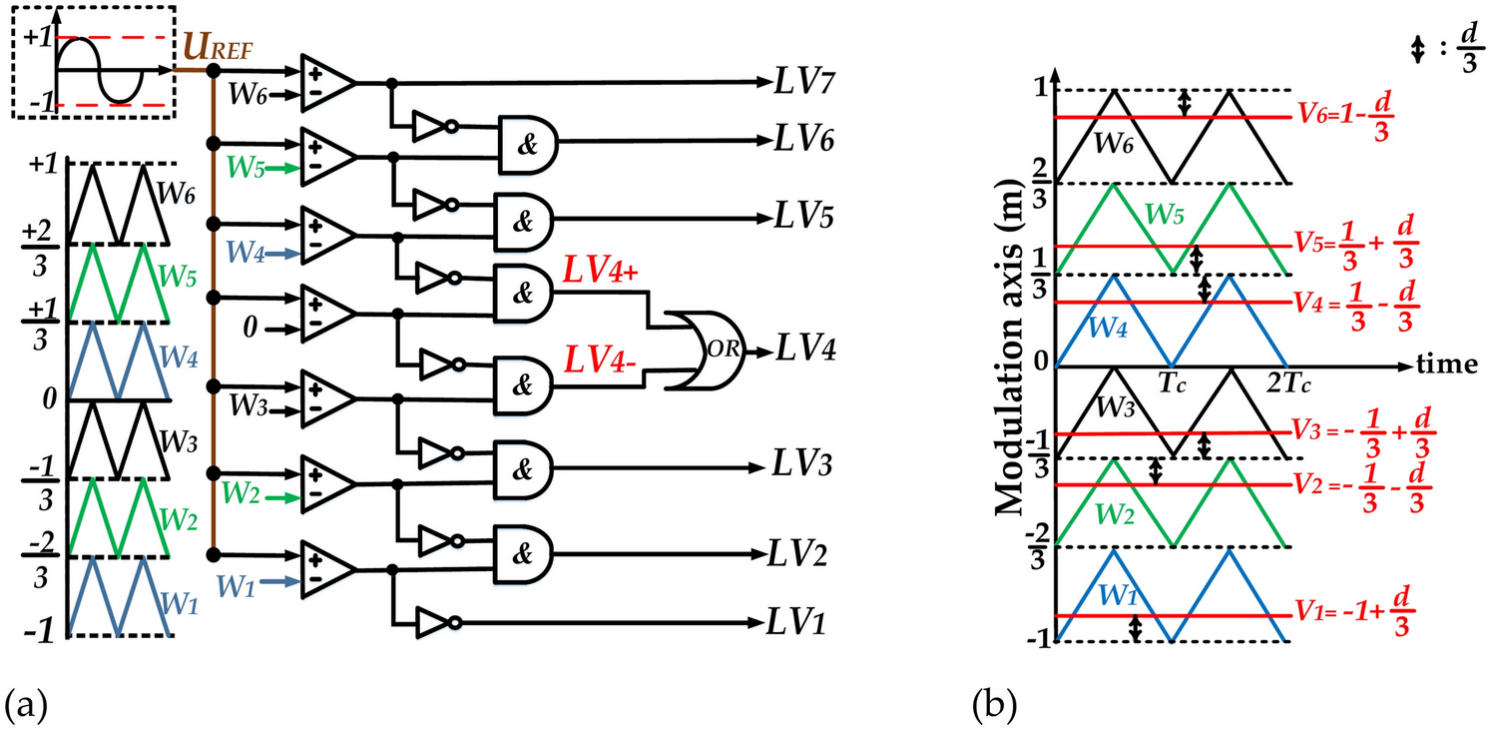
Specific SPWM technique for the qZ-SC7LI, (**a**) Voltage levels developing method, and (**b**) ST states developing method.

As previously mentioned, a modified Level-Shifted SPWM switching strategy is employed to provide the gate signals. Similar to conventional method, seven voltage levels (labeled as *LV*_*1*_ to *LV*_*7*_) are developed by comparing reference voltage with six carrier signals (labeled as *W*_*1*_ to *W*_*6*_), as depicted in Fig. 5a. In order to develop sufficient opportunity to create the ST states for the proposed topology, there is a need to modify the modulation method. Hence, to define the duration in which ST states occur, seven logical-conditional relations are considered. These relations involve comparisons of the carrier signals with specific values, *V*_*1*_ to *V*_*6*_, which are expressed in terms of the ST duty-ratio *(d)*. The mentioned given values are shown by red lines in Fig. 5b.

**Fig. 6:**
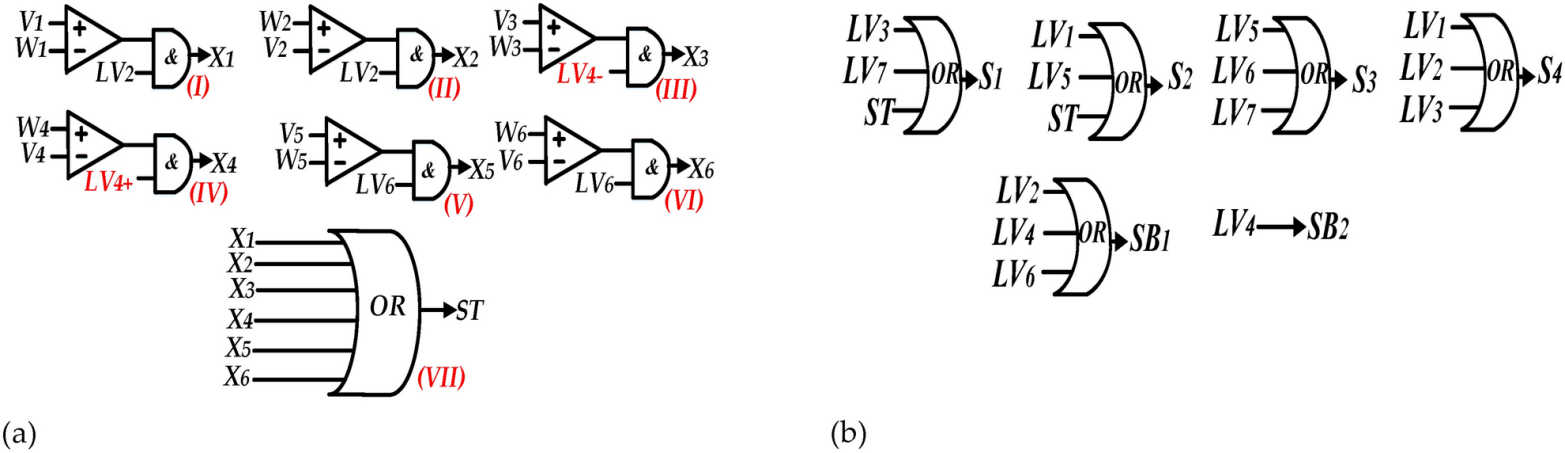
Logic schematics, (**a**) Logic schematic for ST state developing, and (**b**) Logic schematic to generate gate signals.

The aforementioned logical-conditional relations are expressed by (16) and illustrated by Fig. 6(a).

16 $$\begin{gathered} {\text{X}}_{{1}} = if\left( {V_{1} = - 1 + \frac{d}{3} > W_{1} } \right) \otimes LV_{2} \, \,\,\,\,\,{\text{(I)}}\,\,\,\,\,{\text{ X}}_{2} \, = if\left( {W_{2} > V_{2} = - \frac{1}{3} - \frac{d}{3}} \right) \otimes LV_{2} \, \,\,\,{\text{(II)}} \hfill \\ {\text{X}}_{{3}} = if\left( {V_{3} = - \frac{1}{3} + \frac{d}{3} > W_{3} } \right) \otimes LV_{4 - } \, \,\,\,\,\,\,(III)\,\,\,\,{\text{X}}_{4} = if\left( {V_{4} = \frac{1}{3} - \frac{d}{3} < W_{4} } \right) \otimes LV_{4 + } \, \,\,\,{\text{(IV)}} \hfill \\ {\text{X}}_{{5}} = if\left( {W_{5} < V_{5} = \frac{1}{3} + \frac{d}{3}} \right) \otimes LV_{6} \,\,\,\,\,\,\,\,\,\,\,\,\,\,\,\,\,(V)\,\,\,\,\,{\text{ X}}_{{6}} = if\left( {V_{6} = 1 - \frac{d}{3} < W_{6} } \right) \otimes LV_{6} \,\,\,\,\,\,\,\,(VI) \hfill \\ {\text{ST = X}}_{{1}} \, \oplus {\text{ X}}_{{2}} \, \oplus {\text{X}}_{{3}} \, \oplus {\text{X}}_{{4}} \oplus {\text{X}}_{{5}} \oplus {\text{X}}_{{6}} \,\,\,\,\,\,\,\,(VII) \hfill \\ \end{gathered}$$

Ultimately, logic-schematic to generate gate signals for switches is exhibited by Fig. 6b.

**Fig. 7:**
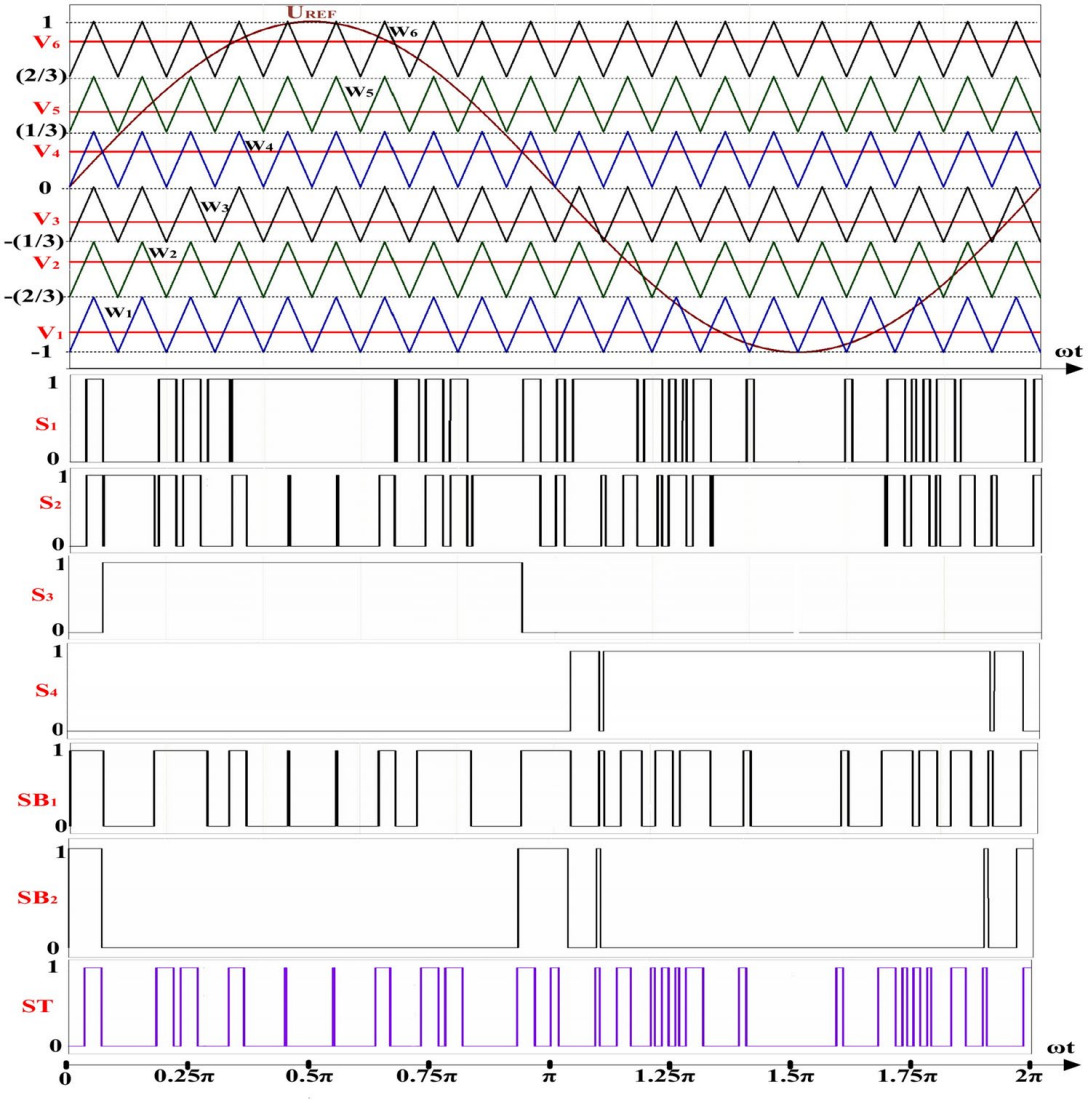
Gate signals generated from proposed modified modulation strategy.

The gate signals produced from the proposed modified modulation strategy, along with ST duration are depicted in Fig. 7.

As inferred from Fig. 7, the noteworthy aspect in this modulation is the relatively more uniform distribution of ST states over a fundamental period *(T)* compared to the previously presented modulation methods. This enhances the precision of voltage and current balance relations used in the mathematical modeling of qZS network, thereby aligning them more closely with real condition.

Additionally, from the perspective of circuit performance improvement, the key feature of this method is ability to create ST states at each switching period *(T*_*c*_*)*. As a result, this method can generate ST states not only at *LV*_*4*_ but also at *LV*_*2*_ and *LV*_*6*_, leading to a reduction in voltage ripple of the switched capacitor (*Δv)* of the inverter output voltage *(V*_*out*_*)*. Another consequence of the mentioned feature is an increase in charging duration of capacitors *C*_*5*_ and *C*_*6*_ resulting in lower *Δv* and smoother inrush current in charging state.

To provide further clarity, Eqs. (17–18) illustrate the relationship between the voltage ripple of the switched capacitors (*C*_*5*_ or *C*_*6*_) and their inrush current:

17 $$\Delta v_{c5(or6)} = \frac{{I_{LD} }}{{C_{5(or6)} }}\Delta t$$

18 $$I_{inrush} = \frac{{\Delta v_{c5(or6)} }}{{R_{Path} }}$$

where Δv_c5 (or6)_, I_LD_, *C*_*5(or6)*_, Δt , I_inrush_, R_path_ represent voltage ripple of capacitors (*C*_*5*_ or *C*_*6*_), load current, capacitance of the capacitors(*C*_*5*_ or *C*_*6*_), discharging duration of capacitors(*C*_*5*_ or *C*_*6*_) , the total resistance of charging path capacitors (*C*_*5*_ or *C*_*6*_), and inrush current respectively.

According to these equations, a shorter discharging duration results in a smaller voltage ripple. Consequently, this reduction in voltage ripple leads to a decrease in inrush current. Referring to Fig. 7, it is evident that the switched capacitors (*C*_*5*_, *C*_*6*_) are charged during each switching period. This results in a shorter discharging period and, therefore, a reduced inrush current.

### Passive component selection

In the AC-side of the proposed topology, the key considerations for selecting passive elements revolve around two critical factors: the inrush current and the allowable voltage drop of capacitors *C*_*5*_ and *C*_*6*_. In the proposed circuit structure, beyond the resistances in the charging path, the charging current of capacitors is constrained by the inductors of the qZS network.

Clearly, the voltage drop of capacitors *C*_*5*_ and *C*_*6*_ depend on the grid (or load) current and the total discharge time of capacitor (*C*_*5*_ or *C*_*6*_) during one fundamental period *(T)*, so below estimation can be considered for selecting of the capacitance values:

19 $$\Delta v_{{c_{5} }} = \Delta v_{{c_{6} }} = \frac{{m{\text{I}}_{gm} t_{d} }}{{C_{5(or6)} }}$$

where *I*_*gm*_, *t*_*d*_*, Δv*_*c5,*_ and *Δv*_*c6*_ are peak value of grid current and, the total discharge time of capacitor (*C*_*5*_ or *C*_*6*_) during one fundamental period (T), the voltage drop of capacitor *C*_*5,*_ and the voltage drop of capacitor *C*_*6*_*,* respectively, in (19).

Assuming a predetermined voltage drop for the mentioned capacitors *(Δv*_*c*_*),* their capacitance can be estimated as:

20 $$C_{5} = C_{6} = \frac{{m{\text{I}}_{gm} t_{d} }}{\Delta vc}$$

According to Fig. 4, the discharge durations of *C*_*5*_*, C*_*6*_ charging corresponds to the negative and positive half cycles, respectively.

In order to estimate value of *C*_*1*_ to *C*_*4*_, which are discharged to *L*_*1*_ to *L*_*4*_ during ST state operation, following relation can be applied:

21 $$\, (C_{1} \Delta v_{c1} + C_{2} \Delta v_{c2} ) = \frac{{P_{o} d}}{{2f_{c} V_{in} }}$$

where *Δv*_*c1,2*_, *V*_*in*_, and *P*_*O*_ are voltage drop of *C*_*1*_*,*_*2*_, the voltage of input DC source, and output power of the inverter, respectively. To ensure that the voltage ripple of the capacitors is within the permissible range, the following relations should be considered:

22 $$C_{1} = C_{4} > \frac{{d(1 - 2d)P_{O} }}{{2(1 - d)\beta_{1} f_{C} V_{in}^{2} }},C_{2} = C_{3} > \frac{{(1 - 2d)P_{O} }}{{2\beta_{2} f_{C} V_{in}^{2} }}$$

where *β*_*1*_represents the allowable voltage ripple ratios of *C*_*1, 4*_, and *β*_*2*_ indicates the permitted voltage ripple ratios of the capacitors *C*_*2, 3*_, respectively, as mentioned in (22).

In order to ascertain inductance for *L*_*1*_ to *L*_*4*_, below relation is regarded:

23 $$\Delta v_{l1,\max } = L_{1,des} \frac{\sigma }{\tau } \, \Rightarrow L_{1,des} = \frac{{\Delta v_{l1,\max } \tau }}{\sigma }$$

where *Δv*_*l1,max*_*, L*_*1,des*_*, σ*_*,*_* τ* are maximum voltage ripple across *L*_*1*_*,* estimated value of *L*_*1*_*,* permitted value for variation of current flowing through *L*_*1*_*,* and maximum interval between two adjacent ST states. Assuming a constant input voltage, and by having predetermined values for *d*, *f*_*c*_, and *σ*_,_ appropriate value for *L*_*1,des*_ and *L*_*1*_ to *L*_*4*_ is estimated as:

24 $$\, L_{1,des} = \frac{{V_{in} (1 - d)}}{{f_{c} \sigma (1 - 2d)}} \Rightarrow L_{1} = L_{2} = L_{3} = L_{4} = L_{1,des}$$

In order to estimate the value of the filter inductor *(L*_*g*_*)* for grid-tied applications, the current ripple is regarded as:

25 $$\Delta i_{g} = \frac{{\left( {1.5mV_{DC} - V_{m} \sin \left( {\omega t} \right)} \right)\left( {m\sin \left( {\omega t} \right)} \right)}}{{6L_{g} f_{c} }}$$

where *V*_*m*_, and *Δig* are the peak value of grid voltage, and grid current ripple, respectively. Assuming constant values for *V*_*DC*_ and *V*_*m*_, the maximum amount of current ripple *(Δi*_*g,max*_*)* is:

26 $$\Delta i_{g,\max } = \frac{{m\left( {0.75V_{DC} } \right)^{2} }}{{6L_{g} V_{m} f_{c} }}$$

Assuming the highest allowable current ripple (δ), the interface filter *(L*_*g*_*)* can be calculated as follows

27 $$L_{g} = \frac{{m\left( {0.75V_{DC} } \right)^{2} }}{{6f_{c} V_{m} \delta }}$$

## Voltage across parasitic capacitors & leakage current

The main challenge in transformerless PV inverters is managing leakage current. There is no galvanic isolation between the PV panels and the grid without a linking AC transformer, which can lead to high-frequency leakage currents. These currents can pose safety risks and affect the performance and efficiency of the PV systems. There are several methods to mitigate leakage current which are introduced in references^24,25^. Regarding SC-MLIs, researchers have suggested various topologies in which they use the common ground method to completely eliminate leakage current^26,27^. While the application of this method results in the complete eradication of leakage current issue, its implementation is not feasible for many topologies. Moreover, it disrupts the overall symmetry of the topology.

In the proposed topology, the overall structure is symmetrized by clamping input capacitors and qZS sub-networks to the middle-point (MP). This ensures voltages across the stray capacitors remain constant. Therefore, referring to Fig. 2, it can be observed that average voltages across stray capacitors *(C*_*PV1*_*, C*_*PV2*_*)* are obtained as:

28 $$v_{{pv_{1} }} = \frac{{V_{in} }}{2},v_{pv2} = - \, \frac{{V_{in} }}{2}$$

Consequently, the leakage current flows to the ground due to variation of voltage across stray capacitors, and it can be assessed as:

29 $$\left\{ \begin{gathered} i_{g} = {\text{I}}_{gm} \sin \left( {\omega t} \right) \Rightarrow i_{g} ^{\prime} \approx \left( {\frac{1 - d}{{1 - 2d}}} \right)\left( {{\text{I}}_{gm} \cos \left( {2\omega t - \varphi } \right)} \right) \hfill \\ \Delta v_{pv} = \Delta v_{pv1} = - \Delta v_{pv2} = \left( {\frac{2}{{C_{in} }} + \frac{2}{{C_{in} }}} \right)\left( {\frac{{i_{g} ^{\prime}}}{2\omega }} \right) \hfill \\ \end{gathered} \right.$$

where *i*_*g*_*’* and *φ* represent the equivalent grid current on DC-side of the proposed topology and the phase difference caused by qZS network, respectively. Thus:

30 $$i_{cm} = \left( {C_{pv1} + C_{pv2} } \right)\frac{d}{dt}\left[ {\Delta v_{pv} } \right]$$

31 $$i_{cm} = \left( {\frac{1 - d}{{1 - 2d}}} \right)\left( {\frac{{C_{pv1} + C_{pv2} }}{{C_{in} }}} \right)\left( {{\text{I}}_{gm} \sin \left( {2\omega t - \varphi } \right)} \right)$$

Given that the capacity of the stray capacitors is significantly lower than that of *C*_*in ,*_ and *d* is restricted by a low value, the leakage current is definitely negligible. Additional the symmetric structure of qZS acts an equivalent capacitor, enhancing the capacitive effect of the input capacitors and further limiting the leakage current.

## Power loss & efficiency calculation

Assessing power losses is crucial for estimating costs and efficiency. Hence, this section is dedicated to the calculation of power losses within the proposed topology. Significantly, the provided estimations are based on the premise that power-factor is unity, the modulation index is also at unity, and the switches employed are MOSFETs.

Referring to Fig. 4, the resistance of the conducting path at each voltage level, excluding the ST state, is denoted as:

32 $$R_{ON} = \left\{ \begin{gathered} 2R_{ds(on)} + R_{esr} {\text{ LV}}_{{1}} {\text{,LV}}_{{3}} {\text{,LV}}_{{5}} {\text{,LV}}_{{7}} \, \hfill \\ 3R_{ds(on)} + R_{esr} {\text{ LV}}_{{2}} {\text{,LV}}_{{6}} \hfill \\ 4R_{ds(on)} {\text{ LV}}_{{4}} \, \hfill \\ \end{gathered} \right.$$

where *R*_*ds (on)*_ represents the on state resistance of the switches, and* R*_*esr*_ indicates the parasitic resistance of the capacitors, respectively, as mentioned in (32).

Therefore, the conduction loss of inverter side is estimated as:

33 $$P_{Con(NST)} = 2f\sum\limits_{j = 1}^{7} {\int\limits_{{\delta_{j} }} {R_{ON} i_{g}^{2} \left( t \right)dt \, } }$$

where *f* represents the fundamental frequency, *i*_*g*_*(t)* is the grid current, and *δ*_*j*_ is the time interval associated with the j^th^ voltage level over half of the fundamental period *(T)*respectively, in (33).

To include ST states and charging conditions, and assuming the charging current is double the rated grid current, the following terms should be added to the outcome of (33).

34 $$\Delta P_{Con} = 4f \, d\sum\limits_{j = 2,4,6} {\int_{\delta j} {Rds(on)\left( \sigma \right)^{2} dt \, } }$$

where *σ* represents the value for variation of current flowing through *L*_*1*_ in (34), and can be estimated as follow:

35 $$\sigma = \frac{{V_{in} (1 - d)}}{{f_{c} (1 - 2d)L_{1} }}$$

Therefore, the conduction loss of inverter side is estimated as:

36 $$PCon(inv) = PCon(NST) + \Delta PCon$$

Furthermore, the conduction loss of the qZS network has been estimated using the mathematical modeling presented in Sect. 2:

37 $$\begin{gathered} P_{Con(qZS)} = { 2}V_{DF} I_{L1} + (2(1 - d)R_{l} + \frac{{2d^{2} }}{(1 - d)}{\text{R}}_{esr} )I^{2}_{L1} + 2d(R_{l} + {\text{R}}_{esr} )(I_{L1} + \sigma )^{2} \hfill \\ \, \hfill \\ \end{gathered}$$

In the Eq. (36), *V*_*DF*_, *R*_*esr*_, and *R*_*l*_ represent the following parameters respectively;* V*_*DF*_ is forward voltage of diodes;

*R*_*esr*_ is the capacitors parasitic resistance; and *R*_*l*_ is the inductors parasitic resistance. It should be noted that parameter *I*_*L1*_ has already been determined using the mathematical model of qZS network.

Thus:

38 $$PCon = PCon(inv) + PCon(qZS)$$

To evaluate the switching losses of the switches and diodes, an initial scenario is analyzed where the ST states are absent. The power loss of a MOSFET that is switching throughout a fundamental period can be expressed as follows:

39 $$P_{SW,MOS} = \frac{{f_{c} V_{stress} Igm(t_{ri} + t_{fu} )}}{2\pi }$$

where *f*_*c*_*, V*_*stress*_*, I*_*gm*_*, t*_*ri*_*,* and* t*_*fu*_ represent the carrier signal frequency, the highest stress voltage on a switch, the peak of injected grid current, the rise time of the MOSFET, and the fall time of the MOSFET, respectively, as discussed in (38). The values corresponding to the highest stress voltage for each semiconductor device have been calculated in the comparison section (Sect. 6). For simplicity in calculations, the voltage levels corresponding to the network frequency angles are approximately assumed as follows:

40 $$\begin{gathered} \omega t < \sin^{ - 1} - 0.986 \Rightarrow LV_{1} , \, \sin^{ - 1} - 0.986 < \omega t < \sin^{ - 1} - 0.645 \Rightarrow LV_{2} {, }\sin^{ - 1} - 0.645 < \omega t < \sin^{ - 1} - 0.317 \Rightarrow LV_{3} {,} \hfill \\ \hfill \\ \sin^{ - 1} - 0.317 < \omega t < 0 \, \Rightarrow LV_{4} - , \, 0 < \omega t < \sin^{ - 1} 0.317 \, \Rightarrow LV_{4} + , \, \sin^{ - 1} 0.317 < \omega t < \sin^{ - 1} 0.645 \, \Rightarrow LV_{5} , \, \hfill \\ \hfill \\ \sin^{ - 1} 0.645 < \omega t < \sin^{ - 1} 0.986 \, \Rightarrow LV_{6} , \, \sin^{ - 1} 0.986 < \omega t \Rightarrow LV_{7} \hfill \\ \hfill \\ \hfill \\ \end{gathered}$$

Therefore, the switching losses in various switches are estimated as follows:

41 $$P_{SW,S1 - 2} = \frac{{f_{c} VgmIgm(t_{ri} + t_{fu} )}}{3\pi (1 - d)}[(0 - sin^{ - 1} - 0.645) + (\sin^{ - 1} 0.645 - 0)] = \frac{{0.149f_{c} VgmIgm(t_{ri} + t_{fu} )}}{(1 - d)}$$

42 $$P_{SW,S3 - 4} = \frac{{f_{c} VgmIgm(t_{ri} + t_{fu} )}}{\pi (1 - d)}[(\sin^{ - 1} 0.317 - \sin^{ - 1} 0) + (\frac{\pi }{2} + \sin^{ - 1} - 0.986) + (\sin^{ - 1} 0.317 - \sin^{ - 1} 0)] = \frac{{0.258f_{c} VgmIgm(t_{ri} + t_{fu} )}}{(1 - d)}$$

43 $$P_{SW,SB1 - SB2} = \frac{{f_{c} VgmIgm(t_{ri} + t_{fu} )}}{3\pi (1 - d)}[(\frac{\pi }{2} + \sin^{ - 1} - 0.986) + (\sin^{ - 1} 0.645 - \sin^{ - 1} 0.317)] = \frac{{0.0571f_{c} VgmIgm(t_{ri} + t_{fu} )}}{(1 - d)}$$

where *V*_*gm*_, and *d* represent the peak voltage of grid, duty ratio of ST state, respectively, in (41–43).Additionally, the switching loss of diodes *D*_*3*_*, D*_*4*_ can be estimated as follow:

44 $$P_{SW,D3 - 4} = \frac{{f_{c} (\Delta v_{c5} )}}{{t_{rr} f}}(\sin^{ - 1} \beta - \sin^{ - 1} \alpha )Q_{RR} ; \, \alpha = 0.317, \, \beta = 0.645 \,$$

where *Δv*_*c5,*_* Q*_*RR*_, and *t*_*rr*_ denote the voltage variation of capacitor *C*_*5*_ (or *C*_*6*_), reverse recovery charge of diodes, and reverse recovery duration time of diodes *D*_*3*_, *D*_*4*_, respectively, as described in (43). Assuming that the voltage variation of capacitor *C*_*5*_* is 10%* of peak value of grid voltage, Eq. (44) can be simplified as follow:

45 $$P_{SW,D3 - 4} = \frac{{0.0378f_{c} V_{gm} }}{{t_{rr} f}}Q_{RR}$$

Therefore the total switching loss can be obtained as follow:

46 $$P_{SW(NST)} = P_{SW,S1 - 2} + P_{SW,S3 - 4} + {\text{P}}_{SW,SB1 - SB2} + {\text{P}}_{SW,D3 - D4}$$

In order to take to account for switching losses associated ST states, the following terms should be considered:

47 $${\text{P}} sw,D(1,2) = \frac{{f_{c} }}{{t_{rr} .f}}{\text{d}}\left( {V_{C2} + V_{C1} } \right)Q_{RR}$$

48 $$P_{SW,ST} = \frac{{f_{c} dVgmIgm(t_{ri} + t_{fu} )}}{6\pi }$$

Eventually the total power losses can be estimated as:

49 $${\text{P}}_{loss} = P_{con} + P_{SW(NST)} + {\text{P}} sw,D(1,2) + \Delta {\text{ P}}_{{{\text{SW}}}}$$

Given the values for *V*_*gm*_*, V*_*in*_*, I*_*gm*_*, d, f*_*c*_ and the characteristics of the components, all terms in the power loss relation (48) can be calculated. Consequently, the efficiency can be estimated as follows:

50 $$\eta = \frac{{0.5V_{gm} I_{gm} }}{{0.5V_{gm} I_{gm} + P_{loss} }}$$

## Simulation and discussion

**Fig. 8:**
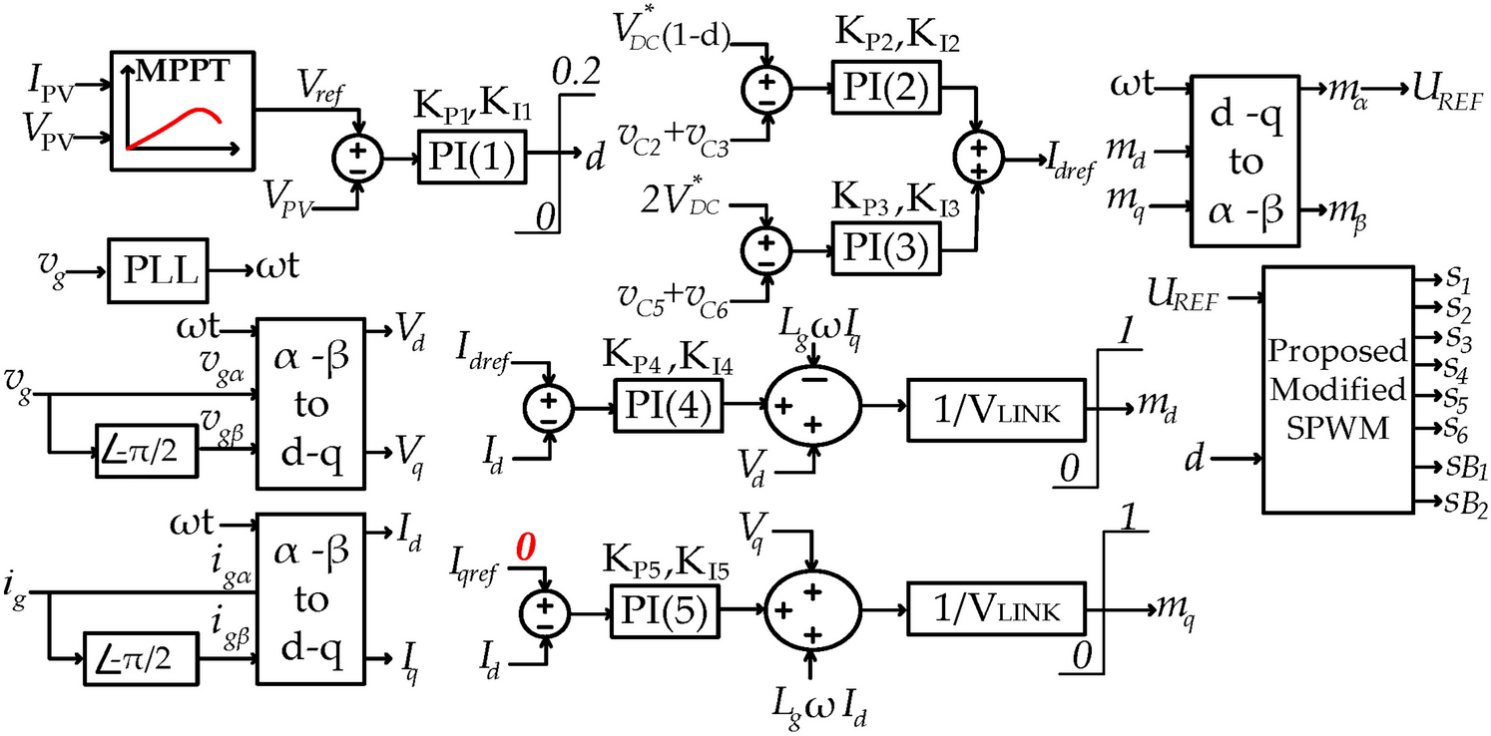
Control scheme of the grid-tied PV system interfaced with the proposed topology at unit power factor.

**Fig. 9:**
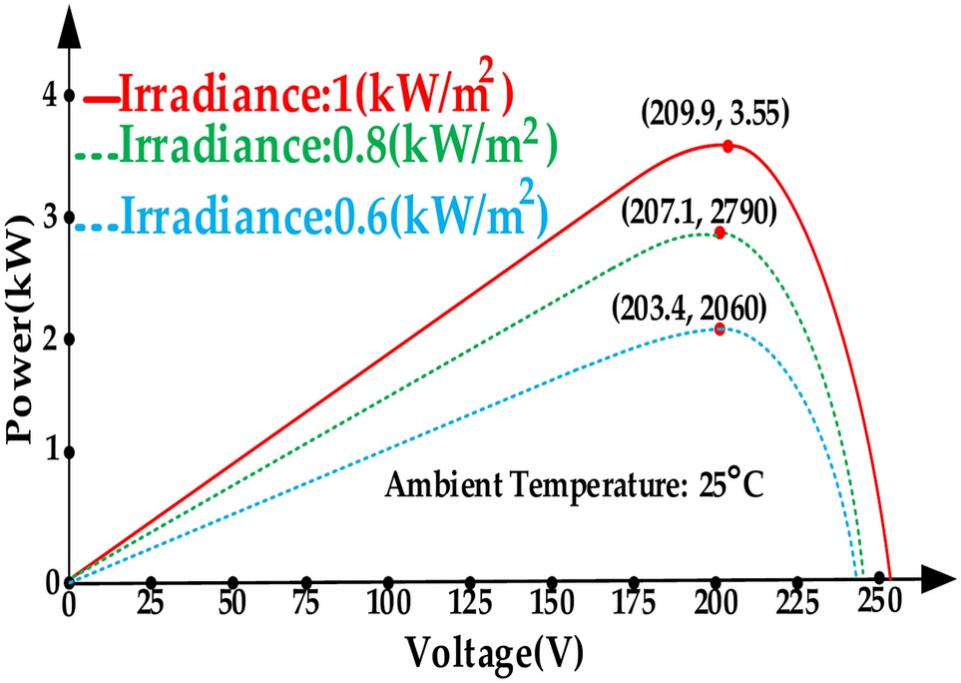
Maximum power point curves of the PV array.

The performance of the proposed topology within a grid-tied PV system is evaluated using PSIM software as a simulation tool in this section. The control scheme for the system at unit power factor is shown in Fig. 8. The maximum power point (MPP) curves of the PV array are depicted in Fig. 9.

In Table 2, the characteristics of the model components and parameters are listed.

**Table 2:** Model components and parameters properties.

Components & parameters	Values
*(L*_*1*_*, L*_*2*_*, L*_*3*_*, L*_*4*_*), L*_*g*_*, R*_*g*_ (related to "*L*_*g*_")	(1,1, 1,1) mH, 2 mH, 0.07* Ω*
*(C*_*in*_*, C*_*1*_*, C*_*2*_*, C*_*3*_*, C*_*4,*_* C*_*5,*_* C*_*6*_*)*	(2,1, 2.2, 2.2, 1, 3.3, 3.3)mF
*R*_*l*_ (Parasitic resistor for all inductors),* R*_*esr*_ (Parasitic resistor for all capacitors)	0.05* Ω*, 0.05* Ω*
*(C*_*pv1,*_* C*_*pv2*_*)*	(300, 300) nF
*R*_*ds(on)*_ (For all switches),*R*_*D(on)*_ (For all diodes)	0.05* Ω*, 0.05* Ω*
*V_*SWF*_ (For all switches), **V_*DF*_ (For all diodes)	0.7 V, 1 V
*v*_*g*_*, *ω	[320 Sin (ωt)] V, 100 π rad/s
(V*_DC_*, V*_*LINK*_*)*,* f*_*c*_	(240, 350) V, 20 kHz
*KP*_*1*_*, KP*_*2*_*, KP*_*3*_*, KP*_*4*_*, KP*_*5*_	− 0.005, − 0.03, − 0.03, 2000, 2000
*KI*_*1*_*, KI*_*2*_*, KI*_*3*_*, KI*_*4*_*, KI*_*5*_	− 0.09, − 20, − 20, 7000, 7000

Descriptions ^*^V_*SWF*_: forward voltage of the MOSFETS, ^**^V_*DF*_: forward voltage of the diodes.

**Fig. 10:**
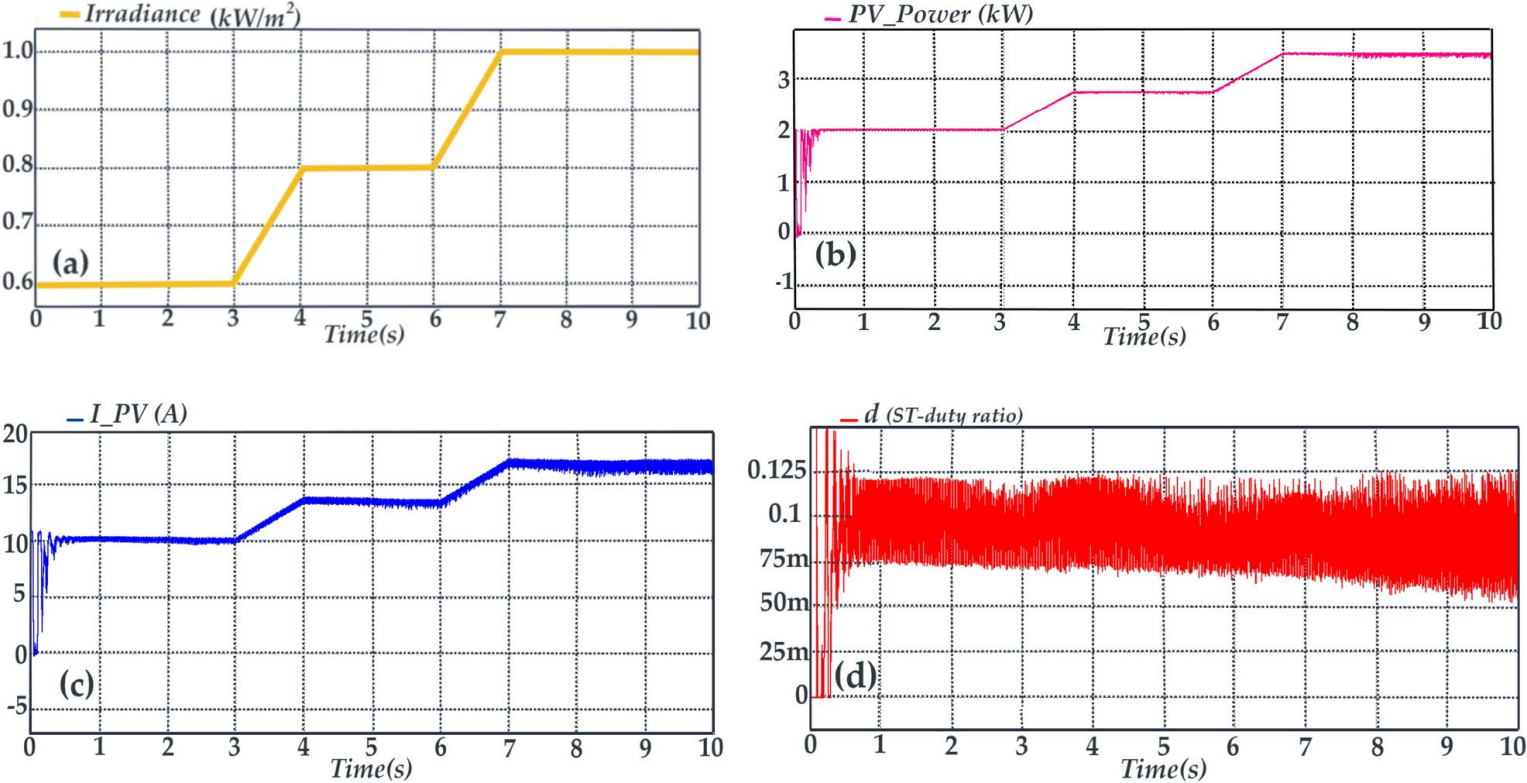
Simulation results, (**a**) Irradiance waveform, (**b**) Extracted power from the PV array, (**c**) Current from the PV array, (d) ST duty-ratio (**d**).

**Fig. 11:**
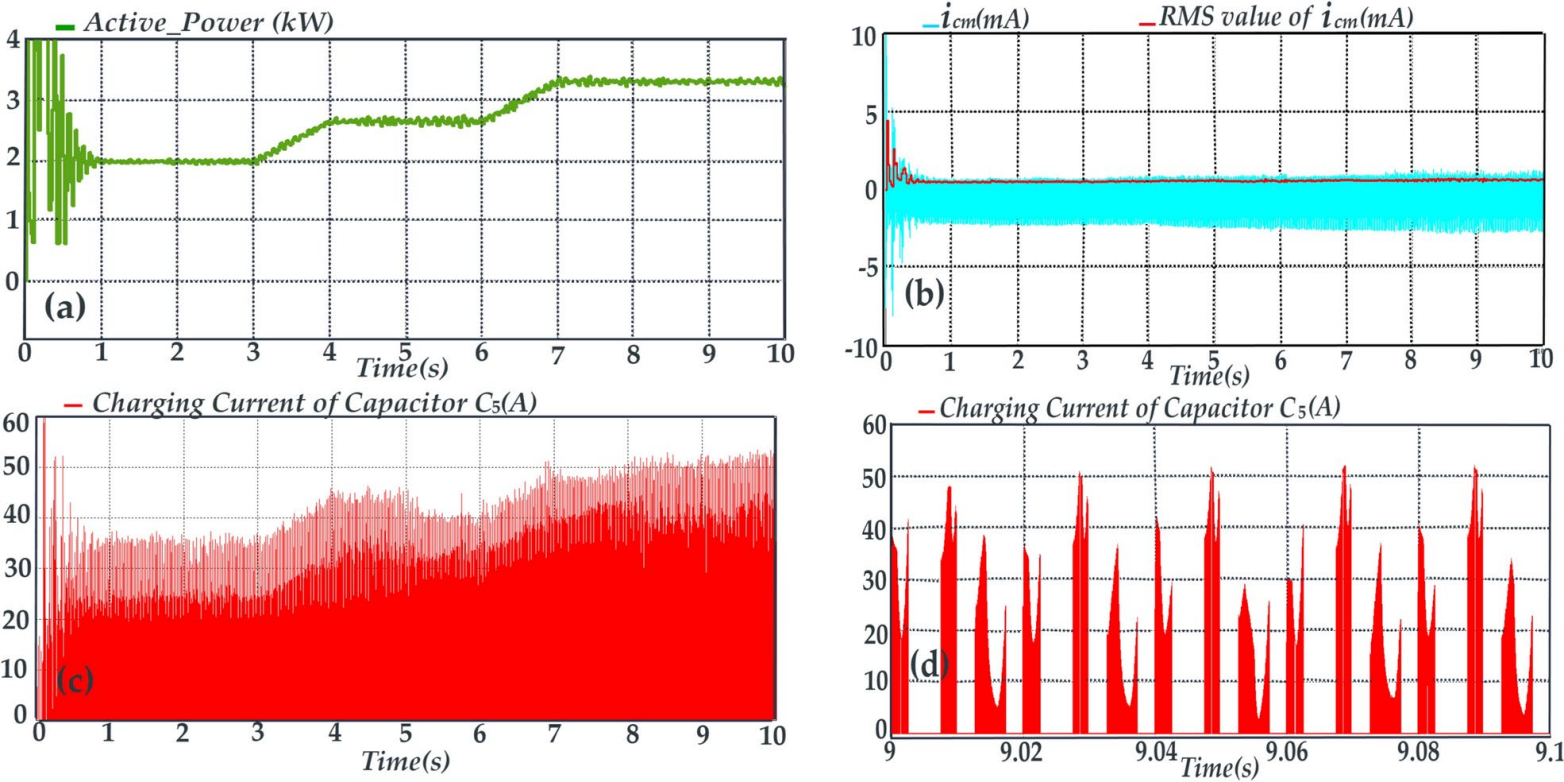
Simulation results, (**a**) Active power, (**b**) leakage current (along with its RMS value), (**c**) charging current of the capacitor *C*_*5,*_ (**d**) charging current of the capacitor *C*_*5*_ during time interval of t = 9 s to t = 9.1 s.

In the control method in Fig. 8, the Perturb & Observe (P&O) technique is used to track the maximum power point (MPP) and the reference of the photovoltaic array voltage (*V*_*PV*_*)* is developed through this technique. It’s worth mentioning that this paper is not aimed at improving MPPT algorithm, hence the (P&O) technique as the most common MPPT algorithm is chosen to evaluate the performance of the proposed topology in a grid tied PV application. Subsequently, by applying a proportional-integral (PI) controller labeled as *PI (1)* to regulate *V*_*PV*_ to its reference value *(V*_*ref*_*)*, the ST duty ratio *(d)* is determined. Referring the Fig. 8, and by the method descripted in reference^28^ parameter *U*_*REF*_ is ascertained. Consequently, gate signals are generated by applying the parameters *U*_*REF*_ and *d* to the proposed SPWM technique. In order to advance the simulation, an irradiance waveform as shown in Fig. 10a is assumed. Thus, the extracted power from the PV array, PV current and ST duty-ratio are shown in Figs. 10b–d, respectively. In Fig. 11a, it is evident that the proposed topology, along with its control system, effectively tracks the maximum power point curves. In Fig. 11b, the peak of the leakage current is below 3 mA and its RMS value is close to zero. These values are far below the rated limits. According to Fig. 11c the charging current of capacitor *C*_*5*_ (current flowing through the diode *D*_*3*_) often is below twice the peak of rated injected current. To provide further clarity, Fig. 11d illustrates the current of capacitor *C*_*5*_ within the time frame spanning from 9.0 s to 9.1 s. According to Fig. 11d, the peak of the charging current is about 2.5 times of the peak of rated injected current. Thus, the charging current is smoothed. The smoothing of the charging current is achieved through the inclusion of inductances *L*_*3*_ and *L*_*4*_ in the charging path, along with the modified SPWM technique, which has the capability to generate shoot-through (ST) states at each carrier period (*Tc*)*.* Due to the symmetry of the proposed topology, this deduction is accepted for the charging current of the capacitor *C*_*6*_ (current flowing through the diode *D*_*4*_). Considering that the inrush current, which represents the most critical current within the proposed topology, passes through switches *S*_*1*_ and *S*_*2*_, as well as diodes *D*_*3*_ and *D*_*4*_, it is imperative that these semiconductor devices are capable of withstanding this current. Therefore, the selection of these semiconductor devices should be done with caution, taking into account the inrush current.

**Fig. 12:**
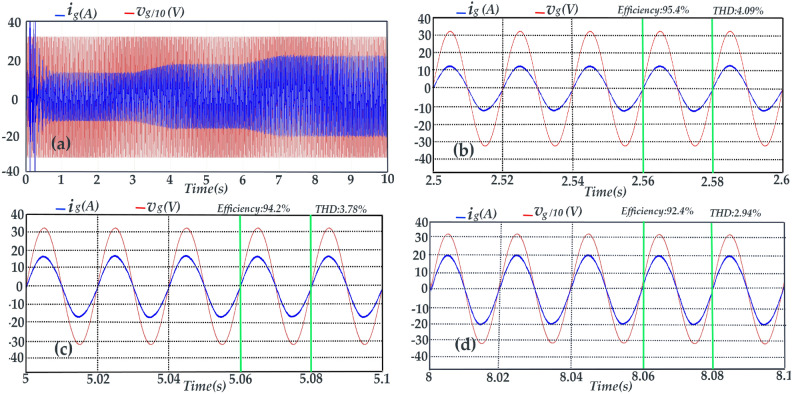
Simulation results of the grid voltage scaled by o.1 and the injected current to the grid during time intervals of (**a**) t = 0 s to t = 10 s, (**b**) t = 2.5 s to t = 2.6 s, (**c**) t = 5 s to t = 5.1 s, and (**d**) t = 8 s to t = 8.1 s.

**Fig. 13:**
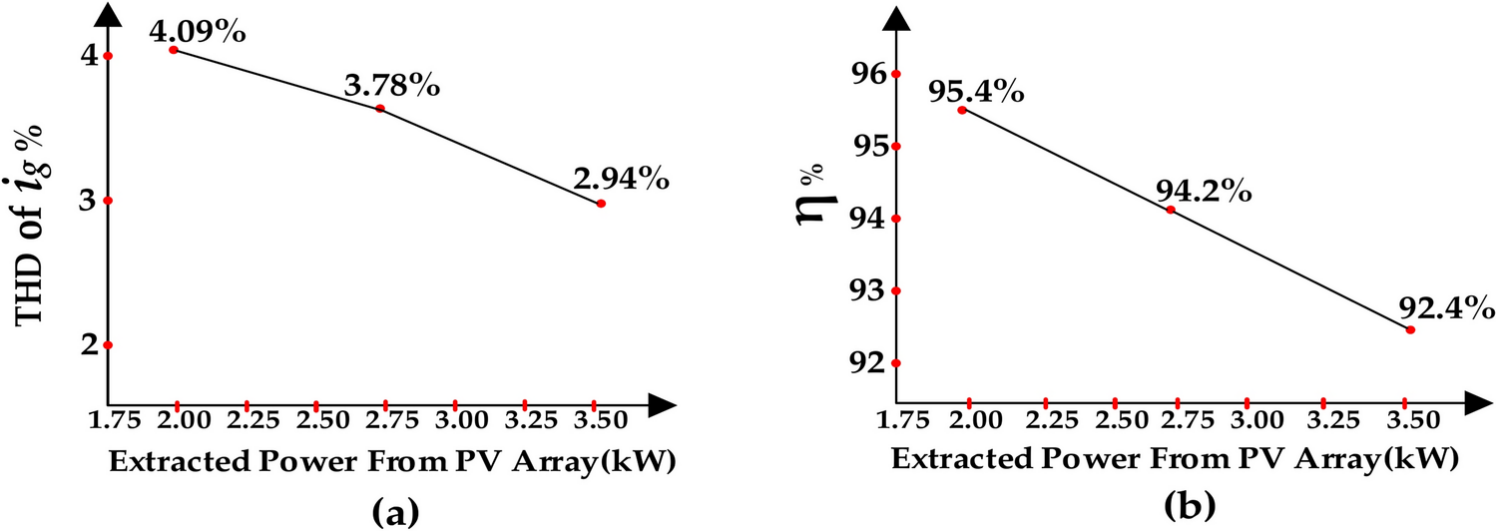
THD and Efficiency (η) results (**a**) THD values of the injected current (i_g_) versus the extracted power from the PV array. (**b**) The efficiency (η) versus the extracted power from the PV array.

The grid voltage scaled by 0.1 and the injected current to the grid are shown in Fig. 12. Referring to this figure, the total harmonic distortion (THD) of the injected current for different irradiation condition is indicated. The THD values of the injected current against power extracted from PV array are depicted in Fig. 13a. Moreover, the efficiency values of the proposed topology versus the extracted power from the PV array are depicted in Figs. 12b–d and 13b.

**Fig. 14:**
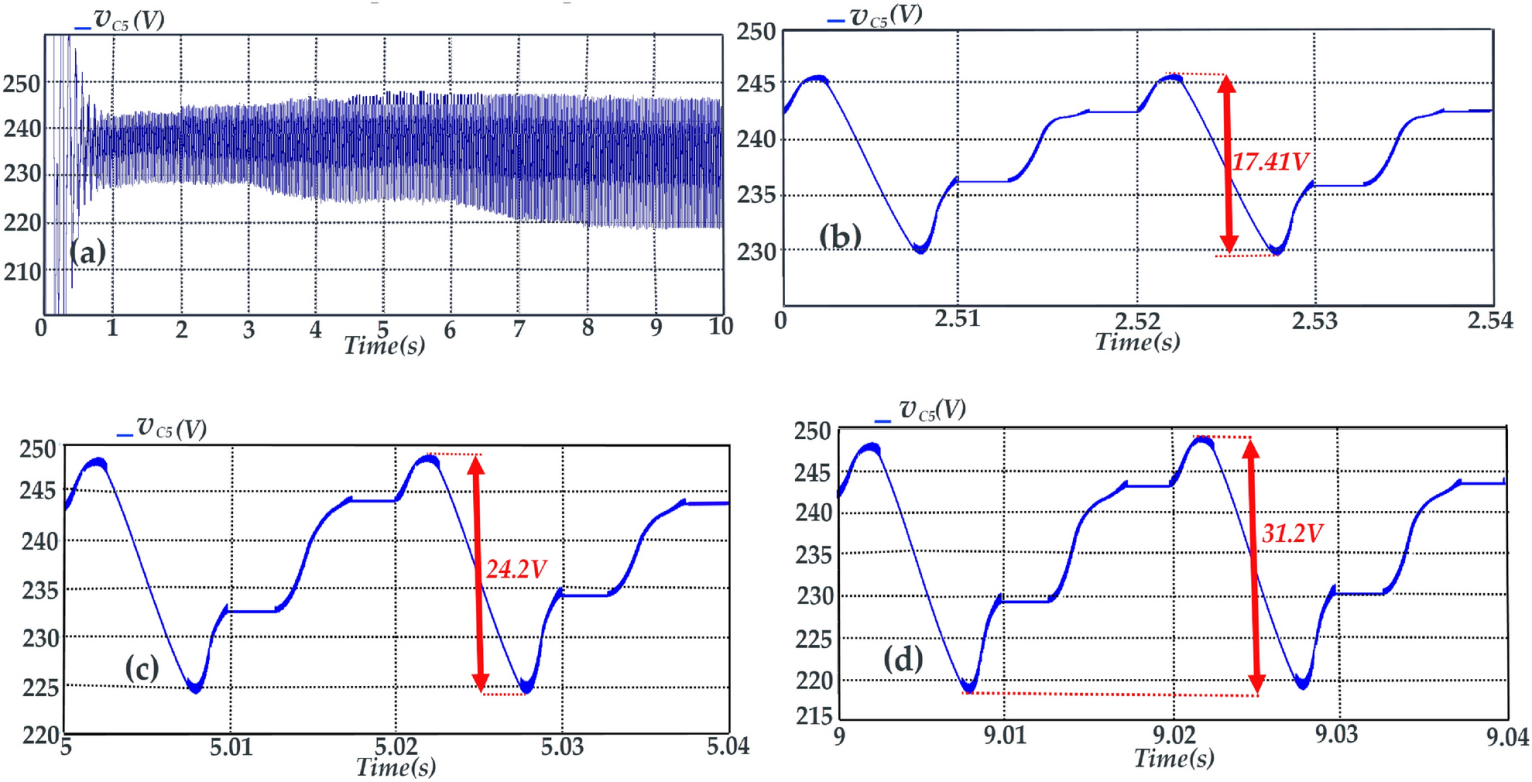
Voltage of capacitor *C*_*5*_ during time intervals of (**a**) t = 0 s to t = 10 s, (**b**) t = 2.5 s to t = 2.54 s, (**c**) t = 5 s to t = 5.04 s, (**d**) t = 9 s to t = 9.04 s.

In Fig. 14, the simulation results of the voltage of the capacitor *C*_*5*_ are depicted. Referring to Fig. 14b–d, the maximum voltage deviations within three time intervals do not exceed the 10% of the nominal voltage of DC-link (*V*_*LINK*_*)*. Therefore, these deviations are considered acceptable. Due to the symmetry of the proposed topology, the recent deduction is accepted for the capacitor *C*_*6*_.

**Fig. 15:**
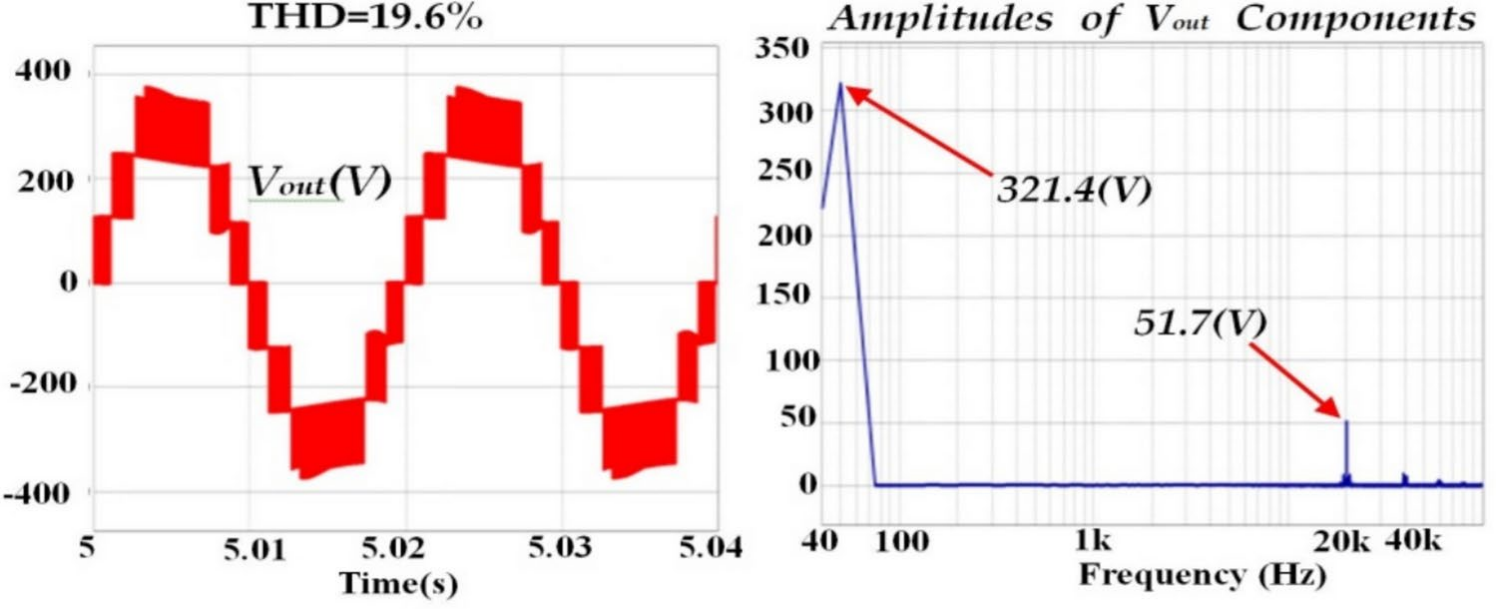
Inverter output voltage *(V*_*out*_*)* and its FFT analysis.

The results of inverter output voltage *(V*_*out*_*)* are presented in Fig. 15. These results indicate the frequency components of *(V*_*out*_*)*, apart from the fundamental frequency, are concentrated around the switching frequency and its harmonics. These components can be effectively filtered out using a compact filter.

## Comparison with pervious topologies

This section provides a concise comparison between the proposed topology (qZ-SC7LI) and recent transformerless single-phase topologies based on ZS/qZS or employing SC-MLI structures.

**Fig. 16:**
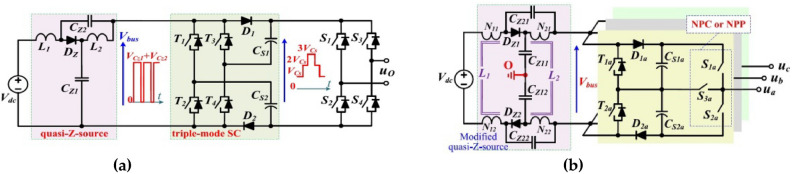
Circuit configurations introduced in reference^21^ (**a**) Circuit configuration of single-phase ZSSC-MLIs (**b**) Circuit configuration of three-phase ZSSC-MLI.

Although reference^21^ introduces a single phase topology, depicted in Fig. 16a, that combines a qZS and a 7-Level SC-MLI, it does not provide any analytical results regarding this configuration. However, due to its asymmetrical structure, this topology cannot inherently suppress leakage current. Additionally, each single-phase unit of the presented symmetric three phase topology in this reference, exhibited in Fig. 16b, cannot be used as a stand-alone single phase PV inverter due to the limitations of its modulation technique and configuration. Actually these limitations stem from the inability of the modulation and configuration to develop ST states for each single phase unit individually. Another issue with this structure is the floating neutral point (point ‘o’ in Fig. 16b) during the creation of ST-states. These issues are addressed in the proposed topology and its specialized modulation technique.

**Fig. 17:**
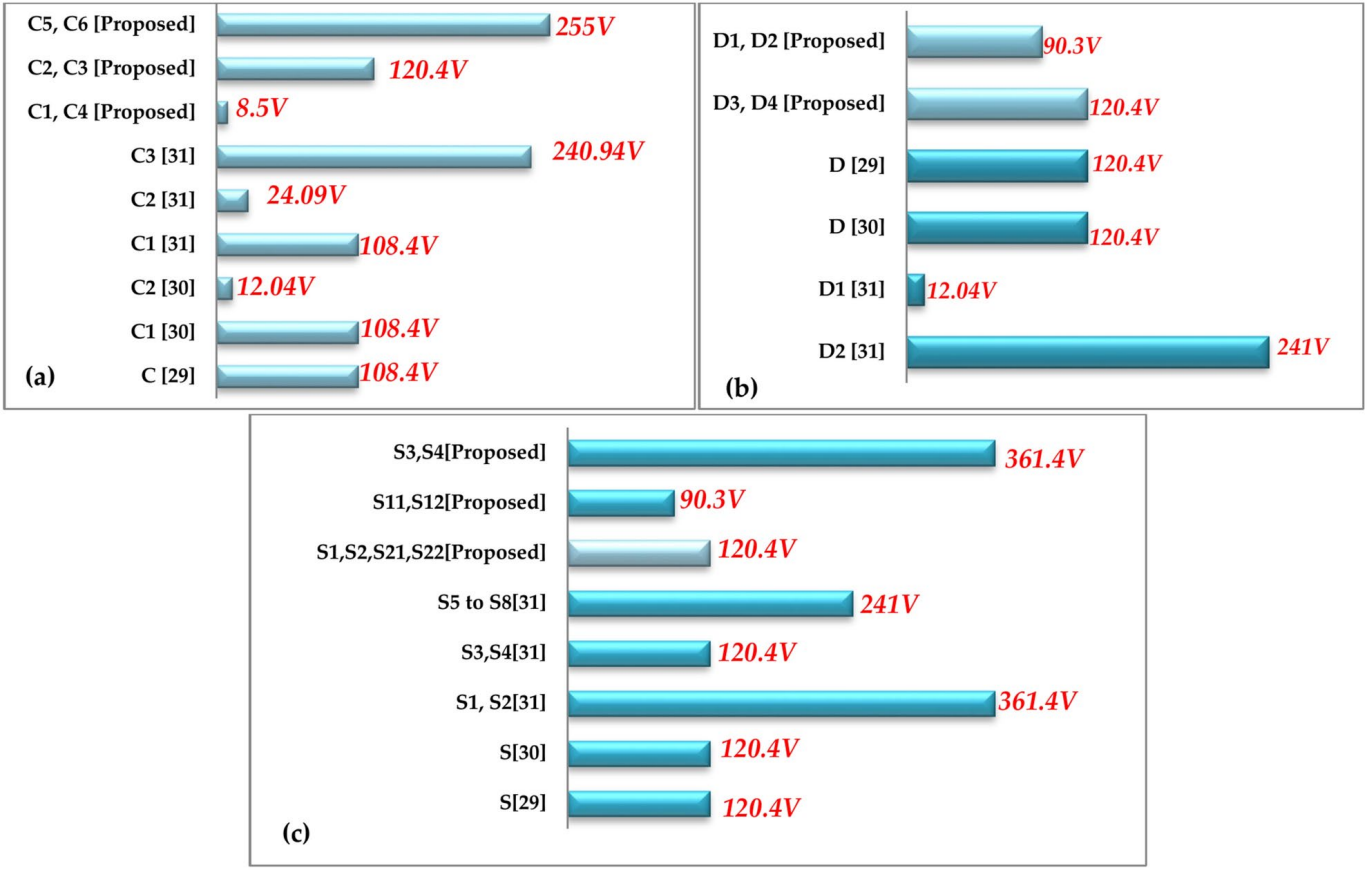
Comparison of voltage stress across capacitors and semiconductors between the proposed topology and other transformerless single-phase 7-Level ZS/qZS-based topologies under the condition of an ST-duty ratio of *d* = *0.1* and an inverter output voltage (RMS) of *230 V*. The comparisons are presented for: (**a**) Capacitors, (**b**) Diodes, (**c**) Switches.

To ensure a direct comparison, the qZ-SC7LI is evaluated alongside state-of-the-art transformerless single-phase 7-Level ZS/qZS-based topologies, as suggested in references^29–31^. Figure 17 illustrates the voltage stresses on the capacitors, diodes, and switches within these topologies for an ST-duty ratio of *d* = *0.1*. The data presented in this figure are assessed assuming inverter output voltage (RMS) is 230 V, adhering to the methodology described in reference^32^.

Additionally, Table 3 provides a comprehensive comparison of the qZ-SC7LI inverter with the topologies proposed in references^29–31^. In this comparison, results are presented by considering the overall boost-factor *(B)*, number of inverter output voltage levels *(N)*, number of switches *(N*_*SW*_*)*, number of diodes *(N*_*D*_*)*, number of utilized inductors*(N*_*L*_*)*, number of utilized capacitors*(N*_*C*_*)*, number of required gate-drivers*(N*_*G-D*_*),* continuity/ discontinuity in input current, voltage stresses across the capacitors, voltage stresses across the diodes, voltage stresses across the switches, total-standing-voltage divided by number of output voltage levels *(TSV/N)*, total harmonic distortion of the inverter output voltage*(THD)*, and Leakage Current Mitigation Ability *(L-C-M-A)*. It is reminded that, the voltage stresses across the capacitors, diodes, and switches in all these topologies are evaluated on a per-unit basis, using the inverter output voltage (RMS) value as the base value. Furthermore Total-Standing-Voltages *(TVSs)* of the topologies are computed based on the relationships presented in reference^33^.

**Table 3:** Comparison of qZ-SC7LI with transformerless single-phase 7-Level ZS/qZS based topologies.

Parameters	ZS-7L^29^	qZS-CHB^30^	DqZS-7LI^31^	qZ-SC7LI (proposed)
B	$1/(1 - 2d)$	$1/(1 - 2d)$	$1/(1 - 2d)$	$1.5/(1 - 2d)$
N_S_	3	3	2	1
N	7	7	7	7
N_SW_	10	12	8	8
N_D_	3	6	2	4
N_L_	6	6	4	4
N_C_	6	6	4	*8
N_G-D_	10	12	8	6
Input current	Discontinuous	Continuous	Continuous	Continuous
Voltage stress across the capacitor	$(\sqrt 2 /3) \,$	$$\begin{gathered} (\sqrt 2 /3){\text{ for }}C_{1} \hfill \\ (\sqrt 2 x.d/3){\text{ for C}}_{2} \hfill \\ \end{gathered}$$	$$\begin{gathered} (\sqrt 2 /3){\text{ for }}C_{1} \hfill \\ (x.d.2\sqrt 2 /3){\text{ for C}}_{2} \hfill \\ (x.2\sqrt 2 /3){\text{ for C}}_{3} \hfill \\ \end{gathered}$$	$$\begin{gathered} (x.d/3){\text{ for C}}_{1} ,C_{4} \hfill \\ (x.\sqrt 2 /3){\text{ for C}}_{{2}} ,C_{3} \hfill \\ (x){\text{ for C}}_{5} ,C_{6} \hfill \\ \end{gathered}$$
Voltage stress across the diode	$$(^{**} x.\sqrt 2 /3) \,$$	$$(x.\sqrt 2 /3) \,$$	$$\begin{gathered} (x.\sqrt 2 /3){\text{ for D}}_{1} \hfill \\ (x.2\sqrt 2 /3){\text{ for D}}_{2} \hfill \\ \end{gathered}$$	$$\begin{gathered} (x.\sqrt 2 /4){\text{ for D}}_{1} ,D_{2} \hfill \\ (x.\sqrt 2 /3){\text{ for D}}_{3} ,D_{4} \hfill \\ \end{gathered}$$
Voltage stress across the switch	$(x.\sqrt 2 /3{\text{ ,x/}}\sqrt 2 )$	$(x.\sqrt 2 /3) \,$	$$\begin{gathered} (x.\sqrt 2 ){\text{ for S}}_{1} ,S_{2} \hfill \\ (x.\sqrt 2 /3){\text{ for S}}_{3} ,S_{4} \hfill \\ (x.2\sqrt 2 /3){\text{ for S}}_{5} \, to \, S_{8} \hfill \\ \end{gathered}$$	$$\begin{gathered} (x.\sqrt 2 /3){\text{ for S}}_{1} ,S_{2} ,{\text{S}}_{21} ,S_{22} \hfill \\ (x.\sqrt 2 /4){\text{ for S}}_{11} ,S_{12} \hfill \\ (x.\sqrt 2 ){\text{ for S}}_{3} ,S_{4} \hfill \\ \end{gathered}$$
***TSV/N	1	0.71	1	0.86
THD	24.05%	29.3%	72.9%	19.6%
****L- C-M-A	NO	NO	YES	YES

*Taking in to account input capacitors, ** x = 1/(1 – d), ***TSV: total—standing—voltage, ****L—C—M—A: leakage current mitigating ability.

According to Table 3, the proposed topology (qZ-SC7LI) demonstrates higher overall boost-factor compared to other topologies. The number of input sources in the proposed topology is limited to one, which is advantageous as it avoids the partial shading PV problem. Additionally, the number of switches used in the proposed structure is fewer than the structures suggested in references^29,30^ and is equal to the number of switches in reference^31^. Furthermore, the number of diodes in the proposed topology is greater than the suggested topologies in references ^29,31^, and fewer than in the topologies recommended in reference^30^. In addition, the number of inductors used in the proposed topology is fewer than the structures suggested in references^29,30^ and is equal to the number of inductors in reference^31^. Although, the number of capacitors used in the proposed topology, including input capacitors, is higher than other topologies, the presence of input capacitors completely eradicates leakage current. Notably, the number of gate drivers required in the proposed topology is less than that in the other topologies presented in this Table, leading to a reduction in the cost of constructing the structure.

According to the literature, input current of the topologies used in photovoltaic application must be continuous to facilitate the implementation of MPPT algorithm, and to reduce stress on the photovoltaic array^34^. As demonstrated in Table 3, this requirement is not met in the topology of reference^29^, but is achieved in topologies of references^30,31^ as well as the proposed topology.

Based on the information presented in Table 3, in the proposed topology, the voltage stresses on capacitors *C*_*5, 6*_ are higher, on Capacitors *C*_*2, 3*_ are approximately equal, and on capacitors *C*_*1, 4*_ are significantly lower compared to the voltage stresses on capacitors in other topologies. Additionally, the voltage stresses on diodes in the proposed topology are less than those of the other suggested topologies. In terms of the voltage stresses on the switches, except for switches *S*_*3*_ and *S*_*4*_, the voltage stresses on the switches in the proposed topology are lower than other topologies. In addition, the maximum voltage stress of the switches in the proposed topology is equal to the suggested topology in reference ^31^ and higher than recommended topologies in reference ^29,30^.

From the perspective of the *(TSV/N)* index as seen in Table 3, the proposed topology performs better than the topologies presented in references^29,31^ but is slightly worse than topology of the reference^30^. Additionally, the proposed topology is in a better position compared to other topologies presented in Table 3 from the point view of *THD* of the inverter output voltage. It is worth mentioning that the high *THD* index in the topology presented in reference ^29^ is primarily due to the method of creating the ST states in this structure. For further explanation, in the mentioned topology, the ST states are only generated at zero-voltage level, so this approach increases *(Δv)* at the inverter output voltage. This issue can have negative effects on the longevity and performance of the semiconductor components, as the components are exposed to high voltage stress levels. This issue is addressed in the proposed topology significantly.

Among the topologies presented in Table 3, only the proposed topology and the suggested topology in reference^30^ have considered solutions for eliminating leakage current, therefore just these topologies can be inverters of choice for non-isolated grid-tied PV application. In addition the method for mitigating leakage current in proposed topology involves maintaining of constant common mode voltage across parasitic capacitor by symmetrizing the overall circuit structure. This method is more straightforward than the method applied in topology of reference^31^. In reference^31^ an L-C-L filter is added to the topology for leakage current suppressing, which complicates the overall circuit structure.

Considering the provided explanations, the proposed topology (qZ-SC7LI) along with its specific modulation method is preferred over other single-phase 7-Level inverters ZS/qZS based presented in Table 3.

**Fig. 18:**
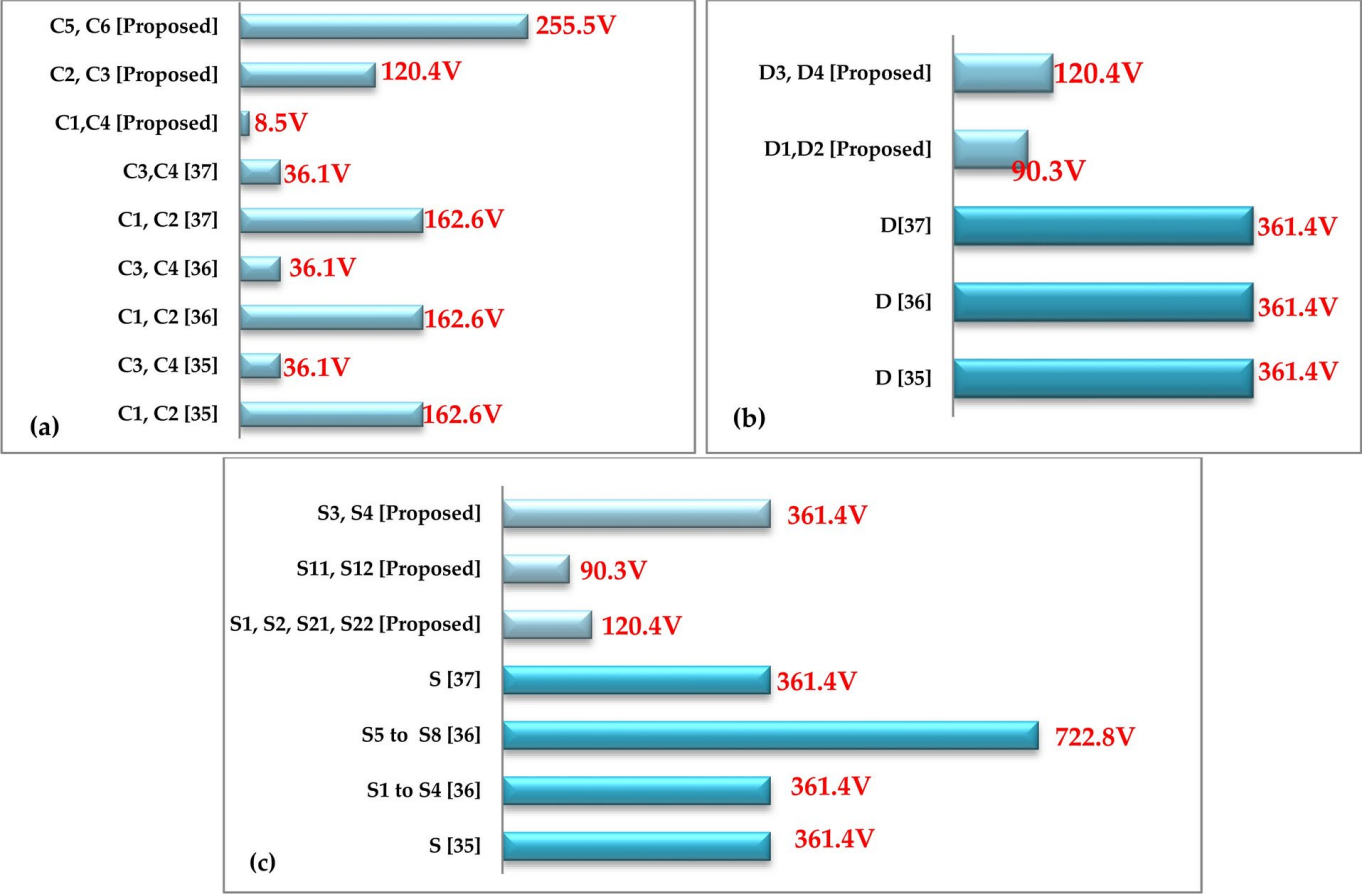
Comparison of voltage stress across capacitors and semiconductors between the proposed topology and transformerless single-phase 5-Level ZS/qZS-based topologies under the condition of an ST-duty ratio of *d* = *0.1* and an inverter output voltage (RMS) of *230 V*. The comparisons are presented for: (**a**) Capacitors, (**b**) Diodes, (**c**) Switches.

Given the scarcity of transformerless single-phase 7-Level ZS/qZS-based topologies in the literature, the proposed topology is also compared with 5-Level qZS-based topologies as suggested in references^35–37^. To provide a quantitative comparison of voltage stress across the capacitors, diodes, and switches in Fig. 18, the corresponding values in the proposed topology are evaluated against those in references^35–37^. Similar to Fig. 17, the data presented in Fig. 18 are evaluated with the assumption that the inverter output voltage (RMS) is 230 V, following the methodology outlined in reference^32^**.**

Furthermore, Table 4 presents a thorough comparative analysis of the qZ-SC7LI inverter and the topologies delineated in references^35–37^. It is important to note the parameters in Table 4 are defined similarly to those in Table 3.

**Table 4:** Comparison of qZ-SC7LI with transformerless single-phase 5-Level qZS based topologies.

Parameters	qZS-NPC^32^	Mqzs-5LI^35^	qZS-5LI^36^	qZ-SC7LI (proposed)
B	$1/(1 - 2d)$	$2/(1 - 2d)$	$1/(1 - 2d)$	$1.5/(1 - 2d)$
N_S_	1	*1*	*1*	*1*
N	5	*5*	*5*	*7*
N_SW_	8	*8*	*8*	*8*
N_D_	6	*3*	*4*	*4*
N_L_	4	*2*	*4*	*4*
N_C_	4	*4*	*4*	*8
N_G-D_	8	*8*	*8*	*6*
Input current	Continuous	Discontinuous	Continuous	Continuous
Voltage stress across the capacitor	$$\begin{gathered} (1/\sqrt 2 ){\text{ for C}}_{1} ,C_{2} \hfill \\ (x.\sqrt 2 .d){\text{ for C}}_{{3}} ,C_{4} \hfill \\ \end{gathered}$$	$$\begin{gathered} (1/\sqrt 2 ){\text{ for C}}_{1} ,C_{2} \hfill \\ (x.\sqrt 2 .d){\text{ for C}}_{{3}} ,C_{4} \hfill \\ \end{gathered}$$	$$\begin{gathered} (1/\sqrt 2 ){\text{ for C}}_{1} ,C_{2} \hfill \\ (x.\sqrt 2 .d){\text{ for C}}_{{3}} ,C_{4} \hfill \\ \end{gathered}$$	$$\begin{gathered} (x.d/3){\text{ for C}}_{1} ,C_{4} \hfill \\ (x.\sqrt 2 /3){\text{ for C}}_{{2}} ,C_{3} \hfill \\ (x){\text{ for C}}_{5} ,C_{6} \hfill \\ \end{gathered}$$
Voltage stress across the diode	$$(^{**} x.\sqrt 2 ) \,$$	$$(x.\sqrt 2 ) \,$$	$$(x.\sqrt 2 ) \,$$	$$\begin{gathered} (x.\sqrt 2 /4){\text{ for D}}_{1} ,D_{2} \hfill \\ (x.\sqrt 2 /3){\text{ for D}}_{3} ,D_{4} \hfill \\ \end{gathered}$$
Voltage stress across the switch	$(x.\sqrt 2 ) \,$	$$\begin{gathered} (x.\sqrt 2 ){\text{ for S}}_{1} - S_{4} \hfill \\ (2x.\sqrt 2 ){\text{ for S}}_{5} - S_{8} \hfill \\ \end{gathered}$$	$(x.\sqrt 2 ) \,$	$$\begin{gathered} (x.\sqrt 2 /3){\text{ for S}}_{1} ,S_{2} ,{\text{S}}_{21} ,S_{22} \hfill \\ (x.\sqrt 2 /4){\text{ for S}}_{11} ,S_{12} \hfill \\ (x.\sqrt 2 ){\text{ for S}}_{3} ,S_{4} \hfill \\ \end{gathered}$$
***TSV/N	1.25	1.25	1.2	0.86
THD	76.4%	38.2%	74.2%	19.6%
****L- C-M-A	NO	NO	NO	YES

*Taking in to account input capacitors, ** x = 1/(1–d), ***TSV: total—standing—voltage, ****L—C—M—A: leakage current mitigating ability.

As shown in Table 4, the overall boost-factor of the proposed topology exceeds that of the recommended topologies in references^35,37^, but falls short of the overall boost factor of the suggested topology in reference^36^.

The number of input sources in all topologies is equal to 1, which is considered a strong point for overcoming the issue of partial shading in photovoltaic applications. The number of switches is the same in all topologies, which is an advantage for the proposed topology because it creates more output voltage levels in the inverter output with the same number of switches. The number of diodes used in the proposed topology is less than that in topology of reference^35^, more than that in topology of reference^36^, and equal to that in topology of reference^37^. The number of inductors utilized in the proposed topology is the same with topologies of references^35,37^ and is more than that of topology suggested in reference^36^. The number of capacitors used in the proposed topology is more than that of all topologies suggested in references^35–37^, which essentially stems from the inherent nature of the switching capacitor topology. The number of gate drivers required in the proposed topology is fewer than in other topologies, which leads to a reduction in construction cost.

According to the information presented in Table 4, the voltage stresses across capacitors *C*_*2*_*, C*_*3*_*, C*_*5*_*,* and *C*_*6*_ in the proposed topology are higher than those in other topologies. In contrast, the voltage stresses across capacitors *C*_*1*_*, C*_*4*_ are significantly lower than those in other topologies. The voltage stresses across the switches and the diodes in the proposed topology are lower than those in other topologies.

Additionally, as seen in Table 4, with regard to *(TSV/N)* and *(THD)* indices, the proposed topology performs better than the other topologies.

Furthermore, among all topologies presented in Table 4, the proposed topology is the only one capable of completely eliminating leakage current.

Based on the given explanations, the proposed topology (qZ-SC7LI), along with its specific modulation method, is favored over the transformerless single-phase 5-Level qZS based topologies presented in Table 4.

In Table 5, qZ-SC7LI is compared with transformerless single-phase SC-MLIs suggested in Refs.^11,21,22^. In this table, the factors *B, N*,* N*_*SW*,_* N*_*D*_,* N*_*L*_, *N*_*C*_, *N*_*S*_, and *N*_*G-D*_ are defined similarly to those in Tables 3, 4. To ensure fair comparisons, the ratios of *N/N*_*S*_, *N*_*SW*_*/N*, *N*_*D*_*/N*,* N*_*L*_*/N*, *N*_*C*_*/N*, and *N*_*G-D*_* /N* also are considered. Furthermore, the abbreviations S-M-S, L-C-M-A and I-R–C-S correspond to the phrases ‘Symmetric Structure’, ‘Leakage Current Mitigating Ability’, and ‘Inrush Current Suppressing’, respectively.

**Table 5:** Comparison of qZ-SC7LI with other transformerless single-phase SCMLIs.

Parameters	Without name^11^	*Single-phase ZSSC^21^	TSC5LI^22^	Asghar^37^	qZ-SC7LI (proposed)
B	1	3/(1-2d)	1.5	1.5	1.5/(1-2d)
N	9	7	5	4	7
N_S_	3	1	1	1	1
N/N_S_	3	7	5	4	7
N_D_	2	3	2	2	4
N_D_/N	0.33	0.43	0.4	0.5	0.57
N_SW_	8	8	8	4	8
N_SW_/N	0.67	1.14	1.6	1	1.14
N_C_	4	4	4	4	**8
N_C_/N	0.44	0.57	0.8	1	1.14
N_L_	0	2	0	0	4
N_L_/N	0	0.28	0	0	0.57
N_G-D_	8	8	8	4	6
N_G-D_/N	0.89	1.14	1.6	1	0.86
S-M-S	Yes	No	Yes	Yes	Yes
L-C-M	Yes	No	Yes	Yes	Yes
I-R–C-S	No	Yes	No	No	Yes

*As shown in Fig. 16. **Taking in to account input capacitors.

As evidenced by Table 5, among the presented topologies, only the suggested topology in refrence^21^ and the proposed topology have an adjustable boost-factor. Additionally, the boost-factor of the suggested topology in Ref.^21^ is higher than that of the proposed topology. In the proposed topology of reference^11^, the boost-factor is equal to 1, meaning this topology does not have the voltage gain enhancement property that exists in SCMLIs.

The number of inverter output voltage levels of the suggested topology in refrence^11^ exceeds those of all topologies presented in Table 5. However, with three input sources, this topology encounters issues related to partial shading and control complexity. Therefore, defining the index *N/N*_*S*_ simplifies the comparison, and in this comparison, the suggested topology in reference^21^ and the proposed topology have a better status compared to other topologies.

In terms of the diodes and inductors used in the topologies in Table 5, the indices *N*_*D*_* /N, N*_*L*_* /N* show that the proposed topology and then the suggested topology in reference^21^ have the highest number of diodes and inductors used relative to the number of voltage levels produced at the inverter output. The elevated values of these indices are attributable to the incorporation of the qZS network. This structure offers advantages such as voltage adjustability and inrush current suppression, which are not available in topologies of references^11,22^.

In terms of the number of switches utilized, index *N*_*SW*_*/N* demonstrates that the proposed topology exhibits superiority over the topology in reference^22^, parity with the topology in reference^21^, and inferiority relative to the suggested topologies in references^11^. Conversely, in the structure of the proposed topology, the inclusion of two bidirectional switches results in a reduction of the required gate drivers by two. Therefore, the index *N*_*G-D*_* / N* of the proposed topology is lower compared to other topologies, leading to a reduction in manufacturing costs.

Moreover, the number of capacitors utilized in the proposed topology exceeds that of other topologies in Table 5, which can be attributed to the implementation of the symmetric qZS network and resultant overall symmetry of the topology. The attribute of symmetry, which is present among the topologies outlined in Table 5, is notably absent solely in the suggested topology in reference^21^. As a result of this feature, the structure inherently has the ability to suppress leakage current. Furthermore, possessing this attribute results in a greater symmetry in the inverter^’^s output voltage waveform. It is noteworthy that without the use of input capacitors and clamping them to the MP point, the proposed topology is capable of significantly damping leakage current. However, the presence of these capacitors and clamping them to the MP point completely eradicates the leakage current.

According to given explanation, among the compared topologies, only qZ-SC7LI simultaneously exhibits five key features as an optimal transformerless single-phase SCMLI in photovoltaic application: boost-factor adjustability, a requirement for a single input source, a symmetric structure, leakage current mitigation, and inrush current suppression capabilities. Additionally, the defined ratios justify the selection of the passive and semiconductor components used in this topology.

## Experimental results

**Fig. 19:**
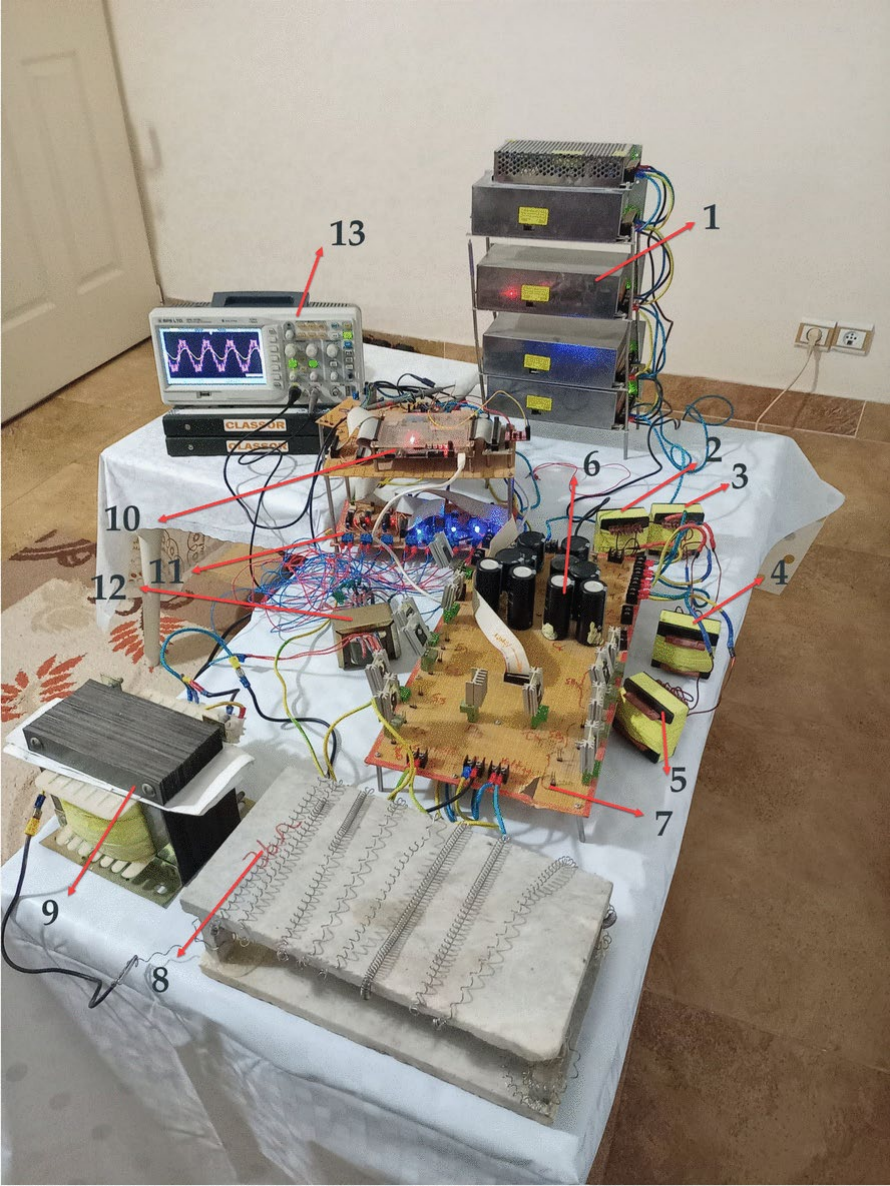
Lab-scaled prototype (1: DC Source, 2: Inductor *L*_*1*_, 3: Inductor *L*_*2*_, 4: Inductor *L*_*3*,_ 5: Inductor *L4*, 6: Capacitors, 7: Montage Power electronics board, 8: Resistive Load, 9: Inductive Load, 10: Digital board (FPGA: Spartan6), 11:Gate drivers(TLP250), 12: Isolating transformer for gate drivers, 13:Digital scope).

**Fig. 20:**
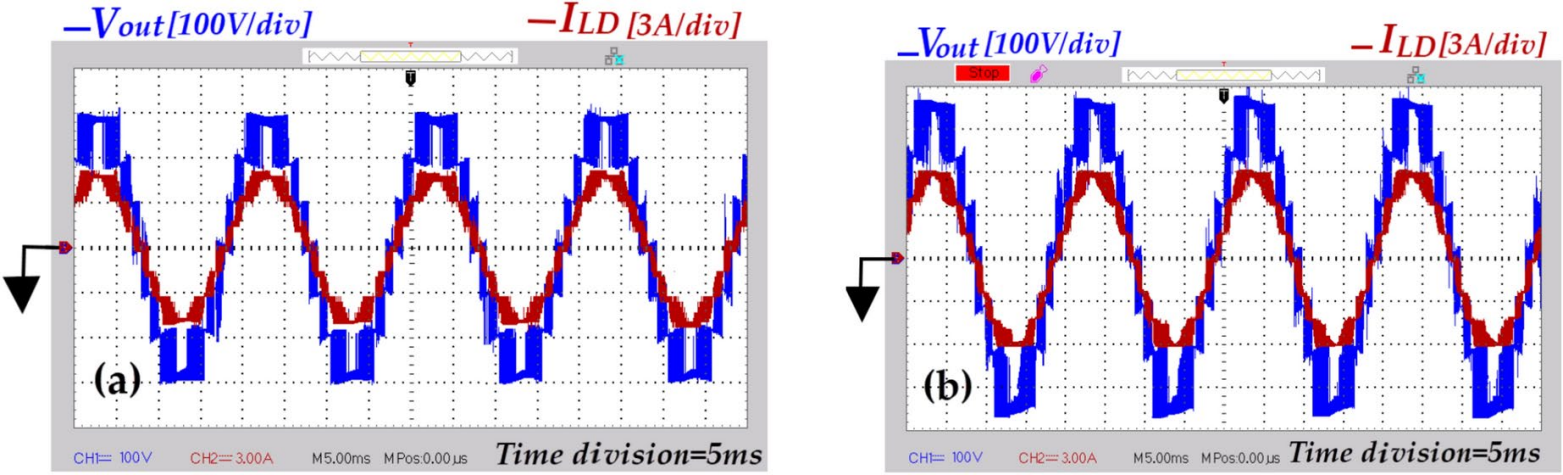
Output voltage and load current under resistive loading [*V*_*out*_ (100 V/div), *I*_*LD*_ (3A/div), Time division (5 ms)], (**a**) Condition where ( *d* = *0*), and (**b**) Condition where( *d* = *0.1*).

**Fig. 21:**
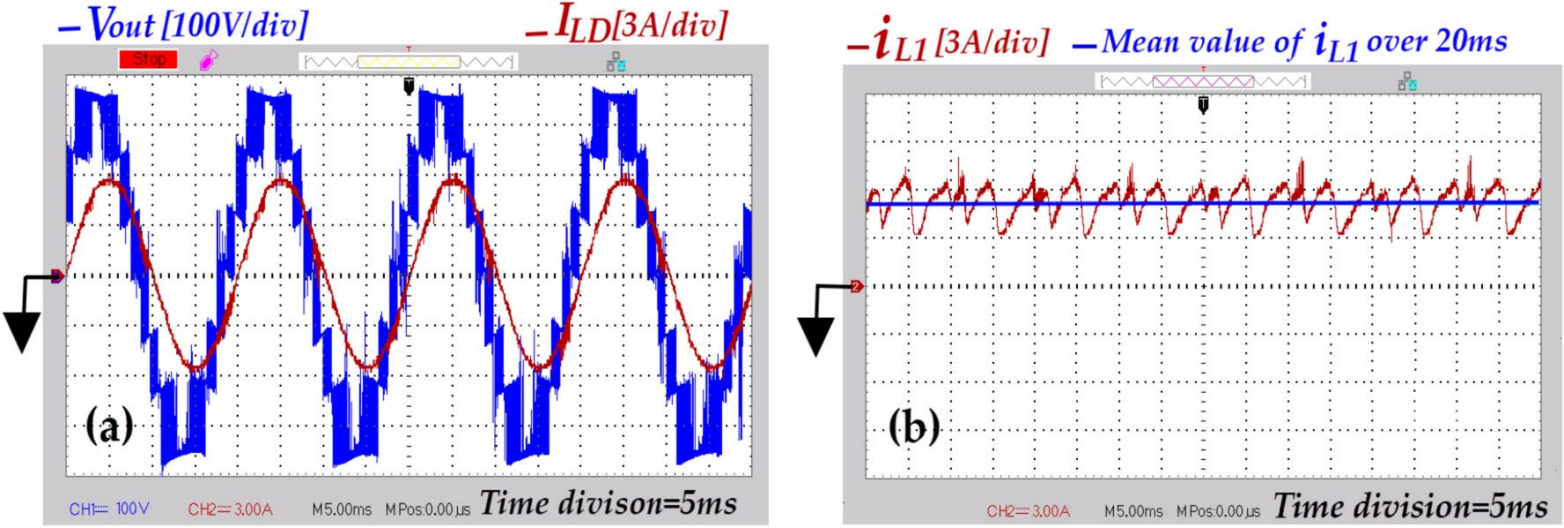
Experimental results under resistive-inductive loading where (*d* = *0.1*), (**a**) Output voltage of the inverter and load current [*V*_*out*_ (100 V/div), *I*_*LD*_ (3A/div), Time division (5 ms)], (**b**) Current flowing through inductor *L*_*1*_ [*i*_*L1*_ (3A/div), Time division (5 ms)].

**Fig. 22:**
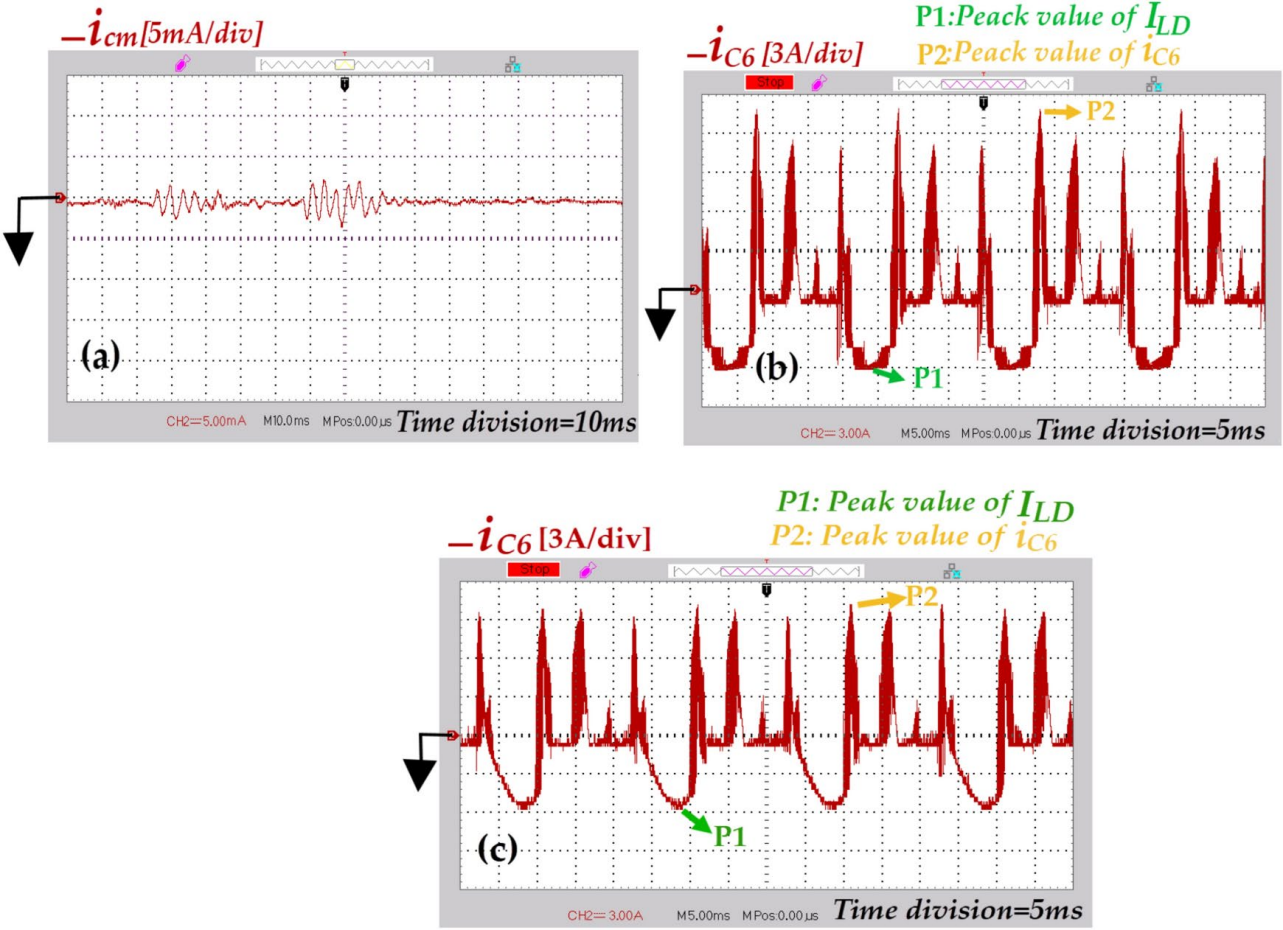
Experimental results where (*d* = *0.1*), (**a**) Leakage current under resistive loading [*i*_*cm*_ (5 mA/div), Time division (10 ms)], (**b**) Current flowing through capacitor *C*_*6*_ under resistive loading [*i*_*C6*_ (3A/div), Time division (5 ms)], and (**c**) Current flowing through capacitor *C*_*6*_ under resistive-inductive loading [*i*_*C6*_ (3A/div), Time division (5 ms)].

**Fig. 23:**
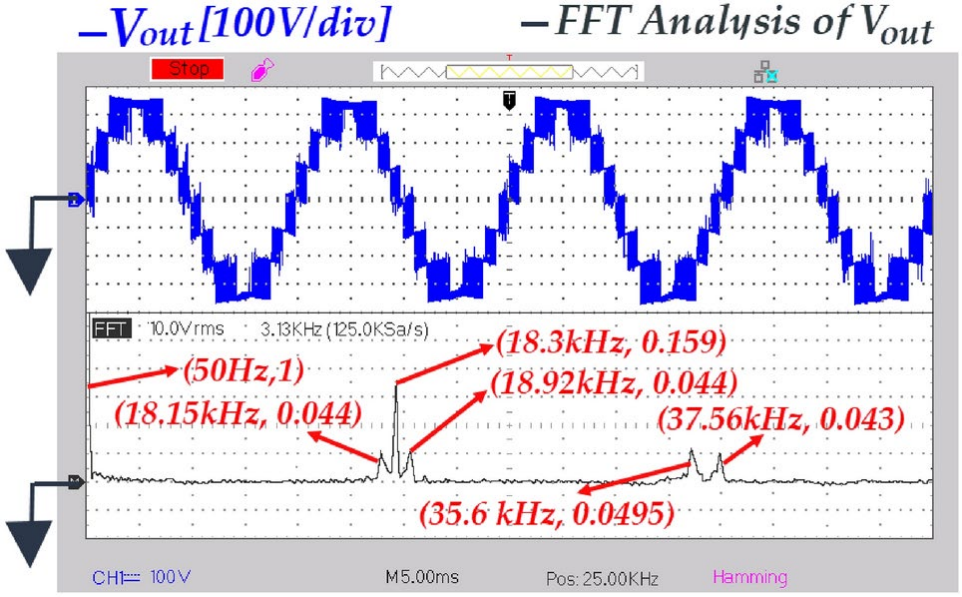
FFT analysis of output voltage of the inverter where (*d* = *0.1*) under resistive loading.

To develop this section, the proposed topology is implemented by regarding components properties listed in Table 6. The employed lab-scaled prototype is exhibited in Fig. 19. As mentioned previously, one of the main features of the proposed topology is the elimination of the leakage current. This aspect is further analyzed by considering two stray capacitors of 150*nF* + 1Ω, which are connected between the point MP and the negative and positive terminals of the DC source. Consequently, the experimental results are depicted in Figs. 20, 21, 22, and 23.

**Table 6:** Components characteristics.

Components	Values or types	Unit
*[L*_*1*_*, L*_*2*_*, L*_*3*_*, L*_*4*_*], R*_*l*_	[1, 1, 1, 1]	*mH*
*[C*_*1*_*,C*_*2*_*,C*_*3*_*,C*_*4,*_* C*_*5,*_* C*_*6,*_ (2*0.5C_*in*_*)], Resr*	[1,2,2,1,2,2,(2*1)], 0.05	*[mF], Ω*
*DC* source*, f*	*200, 50*	*V, Hz*
*m, f*_*c*_	*0.95, 18.3*	*–, kHz*
*R*_*LD*_ (resistive load)	*60*	*Ω*
*L*_*LD*_ (inductive load)	*80*	*mH*
Power switches(MOSFETs)	OSG65R041HZ	–
Diodes	MM80FU040	–
Opto-coupler	TLP250	–
Cores type	E55/28/21	–
Digital board(FPGA)	AX309(SPARTAN6)	–

In Fig. 20a the voltage waveform of the inverter output exhibits a 7-level staircase pattern. Its peak is approximately 1.5 times the input voltage under the condition where (*d* = *0*). Meanwhile, in Fig. 20b the peak value of the output voltage waveform from the inverter increases by about 1.84, under the condition where (*d* = *0.1*). Additionally, the voltage transition from one level to the next is uniform. Furthermore, the transition from the ST-state to NST-state (and vice versa), occurs in just one step, determined by the value of *0.5V*_*DC*_ (half the voltage between points ‘*P*’ and ’*N*’ during NST-state). As a result, there is a reduction of *Δv* at the inverter output.

In order to scrutiny the capability proposed topology in reactive power managing, the resistive-inductive load from Table 6 is connected to the output of the inverter. As depicted in Fig. 21a, the qZ-SC7LI is able to provide the path of reactive power flow. Additionally, in this case the peak value of overall boost-factor is approximately 1.85. In Fig. 21b, the current of the input of the circuit structure(denoted as *i*_*L1*_) remains continiuos, with a mean value over one fundamental period(20 ms) approximately 5.05A. With this input power, the efficiency is 95.2%.

In the following, the inrush current suppression and leakage current mitigation capabilities of the proposed topology are investigated. The related results, are depicted in Fig. 22. The results shown in Fig. 22a demonstrate that the peak of leakage current is approximately 3 mA and its RMS at a frequency of 50 Hz is negligible. These values are well below rated limits (300 mA for peak-to-peak). As observed in Figs. 22b, and c the peak of inrush current resulting from charging of capacitor *C*_*6*_ under resistive, and resistive-inductive loading are approximately 2.5 times, and 2 times the related peak load current. These suppressions occur due two factors: firstly, the presence of the inductors of qZS network in the charging path of the capacitor *C*_*6*_, and secondly the effective performance of the modified modulation strategy. As mentioned previously in Sect. 2, the charging duration of the capacitor *C*_*6*_ occurs in four time intervals. This duration distributes over one fundamental period (20 ms). Additionally, the ST-state can occur in three voltage levels. Thus, the intensity of the inrush current is alleviated effectively. Due to the symmetry of the proposed topology, the recent deduction is accepted for the current flowing through capacitor *C*_*5*_.

To assess the quality of output voltage of the inverter, the FFT analysis (without using any filter at the output terminal) is presented in Fig. 23. According to this figure, the harmonics primarily appeared around the switching frequency and it’s doubled. These harmonics can be removed using a compact sized filter. The THD calculation is carried out according the Fig. 23 as:

51 $$\begin{gathered} THD = \frac{{\sqrt {(0.048)^{2} + (0.159)^{2} + (0.044)^{2} + (0.0495)^{2} + (0.043)^{2} } }}{1} \Rightarrow THD \approx 18.4\% \hfill \\ \hfill \\ \end{gathered}$$

**Fig. 24:**
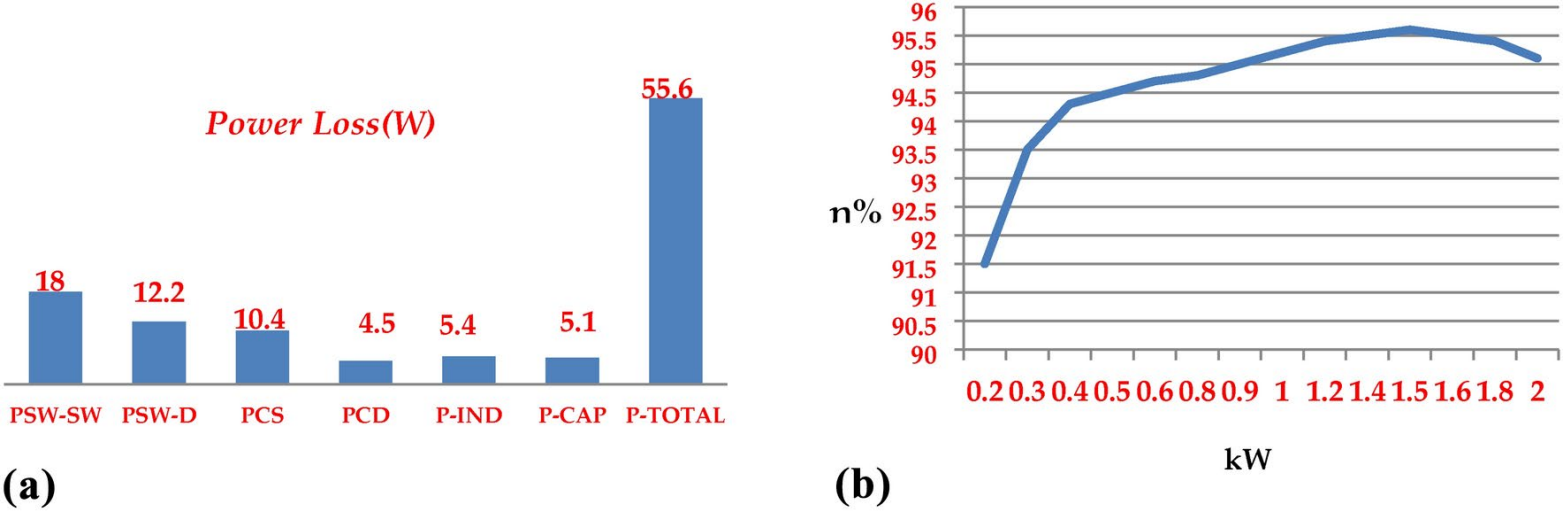
Power loss analysis of a lab-scale prototype includes: (**a**) Distribution of power loss in switches, diodes, inductors, and capacitors at 1.2 kW in the qZ-SC7LI. (**b**) Efficiency (η) versus power in the qZ-SC7LI over a range of 0.1–2 kW under a unity power factor condition and *d* = *0.1*.

To evaluate the efficiency of the proposed topology (qZ-SC7LI), the loss analysis was conducted using the relationships outlined in Sect. 4 and the parameters from Table 6. In Fig. 24a, the semiconductor switching losses *(P*_*SW-D*_*, P*_*SW-SW*_*),* the semiconductor conduction losses *(P*_*CD*_*, P*_*CS*_*)*, the inductors conduction losses (*P*_*IND*_*),* and the ESR losses of the capacitors *(P*_*CAP*_*)* were assessed for an output power of 1.2 kW. These results were obtained from assuming unit power factor, ST duty ratio equal by *d* = *01*. Furthermore, In Fig. 24b the efficiency was assessed for output power in the range of 0.1 to 2 kW under the mentioned assumption. Consequently, an estimated efficiency (η) of 95.4% at 1.2 kW is exhibited by the qZ-SC7LI, as depicted in Fig. 24b.

**Fig. 25:**
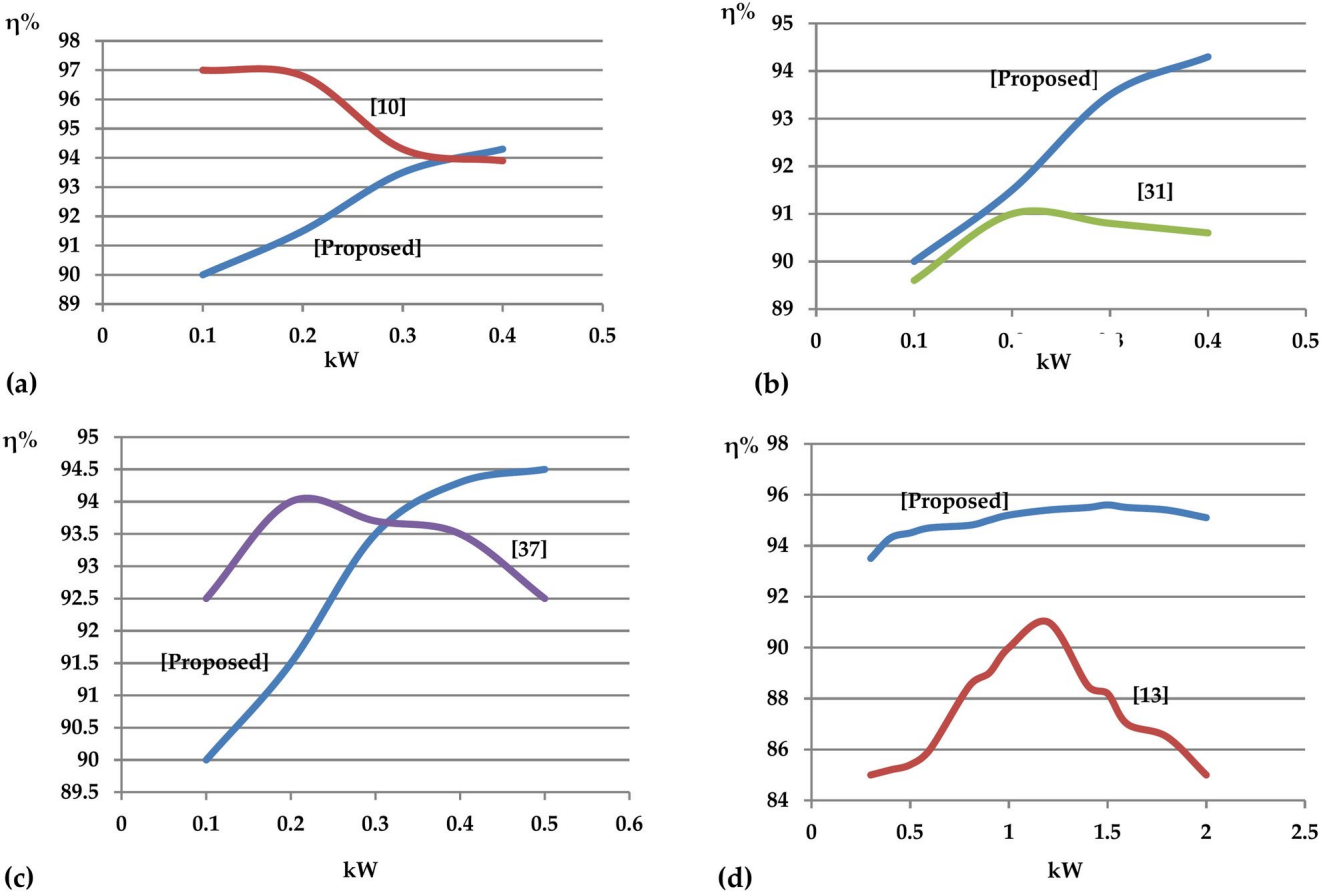
Efficiency comparison of the proposed topology with single phase multilevel qZS based topologies suggested in (**a**) reference^10^, **(b**) reference ^31^, (**c**) refrence^37^, and (**d**) reference^13^.

In the subsequent analysis, the proposed topology is compared in terms of efficiency with other suggested qZS based single phase multilevel inverters topologies. In Fig. 25, the efficiency of the proposed topology is compared with the efficiency of the single phase multilevel qZS based topologies recommended in references^10,13,31,37^. Given the low power range studied in references^10,31,37^, the comparison has been made within low power ranges. However, the strength of the proposed topology lies in its high efficiency at relatively higher power levels, making it suitable for high-power applications. Another significant point is that the proposed topology maintains almost consistent efficiency across the entire output power range of 0.5 to 2 kW. This stability makes it highly suitable for various power levels.

## Conclusions

In this paper, a new topology was proposed by combining a symmetric single-phase SC-MLI with a qZS network, referred to as qZ-SC7LI. Due to its inherent symmetry and access to the middle-point (MP), this topology effectively mitigates leakage current, and ensures the absence of a DC component in the inverter’s output voltage. The qZ-SC7LI increases the voltage gain and makes it adjustable, while ensuring continuous input current, thus facilitating the MPPT technique in photovoltaic applications. Furthermore, the qZ-SC7LI suppresses inrush current by combining the benefits of qZS network and a modified modulation technique. This modified modulation limits inrush current of switched capacitors by shorting the discharging duration. Additionally, the modified modulation reduces the deviation of voltage *(Δv)* in the inverter output by creating ST in three voltage levels. As future work, it is suggested to design and implement a topology using alternative types of Z-source networks on the DC side to reduce the number of passive components. The chosen Z-source network should be compatible with the MPPT algorithm in grid-tied PV applications. The most critical aspect requiring careful consideration is the ability of the employed Z-source network to minimize leakage current.

## Data Availability

All data generated or analyzed during this study are included in this published article.

## Abbreviations

*f*, *ω*, *T*: : Fundamental frequency, *ω* = *2πf*,* T* = *1/f*
*i*_cm_, *f*_c_, *T*_c_: Leakage current, carrier frequency, *T*_*c*_ = *1/f*_*c*_
*T*_*ST*_*, T*_*NST*_: Time interval of shoot-through over one *T*, *T*_*NST*_ = *T-T*_*ST*_
*d*, *G*: ST-duty ratio (*T*_*ST*_*/ T)*, qZS boost-factor
*V*_*out*_*, B*: Output voltage of the proposed topology, Overall boost-factor of the proposed topology
*v*_*lk*_*, v*_*ck*_: Voltage across of kth inductor, Voltage across of kth capacitor
*V*_*Ck*_: Average voltage across of kth capacitor over one fundamental period (T)
*I*_*Lk*_: Average current flowing through kth inductor
*i*_*ck*_: Current flowing through kth capacitor
*V*_*DC*_: Average voltage between points ‘P’ and ’*N*’ over one fundamental period (*T)*
⊗, ⨁: Logical AND operator, logical OR operator
*v*_*g*_*, i*_*g*_*, I*_*LD*_: Grid voltage, injected current to grid, Load current
*m**, I*_*gm*_: Modulation index, Peak value of grid/Load current
